# Fuzzy super twisting mode control of a rigid-flexible robotic arm based on approximate inertial manifold dimensionality reduction

**DOI:** 10.3389/fnbot.2023.1303700

**Published:** 2023-11-09

**Authors:** Xiaoshan Qian, Lisha Xu, Xinmei Yuan

**Affiliations:** ^1^College of Physical Science and Engineering Technology, Yichun University, Yichun, China; ^2^College of Information Science and Engineering, Hunan Women's University, Changsha, China

**Keywords:** rigid-flexible robotic arm, approximate inertial manifold, sliding mode control, super twisting, fuzzy control

## Abstract

**Introduction:**

The control of infinite-dimensional rigid-flexible robotic arms presents significant challenges, with direct truncation of first-order modal models resulting in poor control quality and second-order models leading to complex hardware implementations.

**Methods:**

To address these issues, we propose a fuzzy super twisting mode control method based on approximate inertial manifold dimensionality reduction for the robotic arm. This innovative approach features an adjustable exponential non-singular sliding surface and a stable continuous super twisting algorithm. A novel fuzzy strategy dynamically optimizes the sliding surface coefficient in real-time, simplifying the control mechanism.

**Results:**

Our findings, supported by various simulations and experiments, indicate that the proposed method outperforms directly truncated first-order and second-order modal models. It demonstrates effective tracking performance under bounded external disturbances and robustness to system variability.

**Discussion:**

The method's finite-time convergence, facilitated by the modification of the nonlinear homogeneous sliding surface, along with the system's stability, confirmed via Lyapunov theory, marks a significant improvement in control quality and simplification of hardware implementation for rigid-flexible robotic arms.

## 1. Introduction

Currently, rigid-flexible robotic arms are widely used in industries such as industrial automation, machinery, medical care, and aerospace (Su et al., 2022a; Liu et al., 2023; Zhao and Lv, 2023). How to design robust controllers with high positioning accuracy and repeatability has attracted significant interest from researchers. The rigid-flexible robotic arms utilized in this study offer several notable advantages. Firstly, their adaptability allows them to navigate and operate in complex environments, striking a balance between flexibility and rigidity. This ensures that tasks are executed with a high level of precision without compromising on the structural integrity of the arm (Tavasoli and Mohammadpour, 2018). Furthermore, the unique structure of these arms ensures pinpoint accuracy in control and positioning (Arkouli et al., 2021). Lastly, the rigid-flexible design serves as a buffer to absorb shocks and vibrations, enhancing the durability and reliability of the robotic arm in various applications (Zheng et al., 2022). However, the complexity of the dynamics of rigid-flexible robotic arms and uncertainties caused by unknown parameters, load variations, unmodeled nonlinearities, and external disturbances make this task challenging.

Numerous nonlinear control techniques have been proposed in literature, such as inversion (Alam et al., 2018; Wang et al., 2022), adaptive control (Weiser and Corves, 2019; Zhou et al., 2020), H-infinity control (Rigatos et al., 2016), and Sliding Mode Control (SMC; Bahrami and Rahi, 2003; Hamdi et al., 2015; Kwon et al., 2018). The latter has gained considerable attention recently due to its simplicity and robustness against uncertainties. Its design is based on a surface selected by the user in the system state space and a high-gain switching term that forces system trajectories to converge to and stay on that surface (Hamdi et al., 2015). However, to ensure robustness, rapid finite-time convergence is ensured by choosing a switching gain that is greater than the upper bound of the uncertainty. This excessive choice leads to the well-known chattering phenomenon, which is a major drawback of SMC (Gharooni et al., 2001; Congqing et al., 2013). This interference causes the system actuator to reject high-energy, high-intensity control signals and can lead to degradation or deterioration of the controlled system's mechanical components. To overcome this phenomenon, continuous functions are considered as a replacement for the sign function (Shokouhi and Davaie Markazi, 2018). This method allows for robust and accurate estimation of the uncertain part. However, if the estimation is inaccurate, control performance is affected. Additionally, some literature combines sliding mode with other techniques like fuzzy logic, neural networks, or both (Xu et al., 2010; Rahimi and Nazemizadeh, 2014; Liu et al., 2017; Singla and Singh, 2019). While theoretically powerful, these intelligent methods are computationally complex, potentially making hardware implementation difficult or impossible. Some researchers (Buffinton, 1992; Khalil, 2011; Zarafshan and Moosavian, 2012) have combined first-order SMC with Time Delay Estimation (TDE) methods, which estimate uncertainty and bounded external disturbances without knowing the upper bounds of uncertainty, reducing the choice of switching gain. However, the chattering problem has not been fully resolved and affects convergence time. To eliminate or reduce the chattering phenomenon, higher-order SMC (HOSMC) was introduced by Huston (1981), Kumar et al. (2000), Delgado et al. (2017), and Zhang et al. (2020). Based on this, many algorithms that allow finite-time convergence and reduce chattering, such as suboptimal algorithms, super twisting algorithms, and STA, have been proposed by Kumar et al. (2000) and Grazioso et al. (2019). However, a common drawback of these methods is the correct choice of controller gain, which is essential for achieving finite-time convergence characteristics. Moreover, in the field of rigid-flexible robotic arm control systems, there are few reports on the application of super twisting mode control, making it necessary to analyze the super twisting mode control of rigid-flexible robotic arms to achieve an intelligent and effective control system (Su et al., 2020, 2022b; Qi et al., 2023).

In recent years, fuzzy logic has been widely applied to enhance the efficiency of SMC in controlling uncertain nonlinear systems (Soltanpour et al., 2016). So far, a plethora of fuzzy SMC algorithms have been developed for robotic control systems (Ertugrul and Kaynak, 2000; Huŝek, 2014; Qi et al., 2021; Qi and Su, 2022). In Derbel and Alimi (2006), a hybrid method of fuzzy logic controllers and SMC is given to compute the equivalent control force. The continuous computation of sliding parameters in Javaheri and Vossoughi (2005) improves the performance of robots. The authors in Soltanpour et al. (2013) combine fuzzy logic and SMC to overcome uncertainties and disturbances during robot trajectory tracking. In Khooban and Soltanpour (2013), and Soltanpour and Khooban (2013), optimization techniques are used to optimize the control coefficients of the fuzzy sliding mode system. However, these controllers lack mathematical analysis and closed-loop stability analysis. Applications of FLSMC can be roughly categorized into three types (Tran and Kang, 2015): adaptive fuzzy methods enhance the anti-interference capability of SMC, controller gains are adjusted based on fuzzy logic to alleviate chattering, and discontinuous sign functions are replaced with fuzzy logic during the reaching phase to eliminate chattering. However, the correct selection of initial values for the adaptive fuzzy system is crucial for the rapid convergence of the adaptive law. Benbrahim et al. (2013) proposes an Adaptive Fuzzy Sliding Mode Controller (AFSMC) to estimate the unknown functions needed to overcome existing uncertainties. However, the design of these controllers is quite complex and may pose issues in practical implementation.

While many of these studies have made considerable algorithmic improvements to ensure higher control quality, they haven't maximized the simplicity of controller hardware implementation. To simplify the hardware design of the controller, the model should be reduced in order as much as possible. Currently, one of the better nonlinear dimensionality reduction methods, the Galerkin method (Jefrin Jose, 2014; Muhammad et al., 2014; Nazemizadeh and Nohooji, 2015; Deng et al., 2021; Yuan et al., 2021; Peng et al., 2022; Shang et al., 2022), can effectively reduce the dimension of nonlinear spatiotemporal coupled systems. Still, it entirely ignores fast variables, leading to the loss of some slow variable information integrated with the fast variable system, affecting the model's accuracy. In the dimensionality reduction method based on approximate inertial manifolds (Qiu and Dongya, 2020), fast variables can be expressed through slow variables, so the high-precision approximate model of the original spatiotemporal system can be obtained through fast variable compensation for the slow variable system. Compared with the traditional nonlinear Galerkin method, for systems with uncertain inertial manifolds, approximate inertial manifolds can achieve better dimensionality reduction results. This paper, from the perspective of the reduced model, targets the challenges associated with the poor control quality of the directly truncated first-order modal model of the rigid-flexible robotic arm based on the Galerkin method in Xu et al. (2021b) and the relative complexity of the hardware implementation of the directly truncated second-order modal model controller. We introduce a novel fuzzy super twisting mode control method, which is a significant advancement over traditional approaches, utilizing the approximate inertial manifold dimensionality reduction model of the rigid-flexible robotic arm. First, we innovatively employ a member of the Lipschitz continuous controller family, the super twisting controller (Levant, 1993). By adapting the nonlinear homogeneous sliding surface, we achieve finite-time convergence, which has not been explored in existing literature. Then, a new fuzzy strategy is used to dynamically optimize the sliding surface coefficients in real-time. Compared with the fuzzy method for sliding mode exponent, the adjustment is simpler and easier to implement. Lastly, we solidify the controller's stability through Lyapunov theory, and demonstrate the effectiveness of our breakthrough algorithm via a range of simulations and experiments. In summary, the main contributions of this paper are: (a) a novel fuzzy control strategy, which from the perspective of the sliding surface, offers real-time dynamic optimization of the sliding surface coefficient β. This technique is more streamlined and practical than the existing fuzzy method for the sliding mode exponent, marking a distinct advancement in this field; (b) the incorporation of the super twisting algorithm to revolutionize the reaching law, thereby notably decreasing the chattering phenomenon. This ensures that the tracking error converges rapidly to zero, even in scenarios with external bounded disturbances; (c) the pioneering adoption of the fuzzy super twisting mode control method based on the approximate inertial manifold dimensionality reduction model for the rigid-flexible robotic arm. This approach, when contrasted with the directly truncated first-order and second-order modal models based on the Galerkin method, greatly simplifies the controller's hardware implementation without compromising on the control quality, filling a critical gap in the current research landscape.

The remaining parts of this article is organized as follows: Section 2 delves into the low-dimensional model of the rigid-flexible robotic arm based on approximate inertial manifold. The controller design is detailed in Section 3. Section 4 provides a simulation analysis, encompassing step response analysis, sine wave tracking analysis, and robustness performance. Section 5 offers an experimental verification of the fuzzy super-twisting SMC for the reduced model. Finally, Section 6 concludes the article, highlighting the key findings.

## 2. Low-dimensional model of rigid-flexible robotic arm based on approximate inertial manifold

**Figure 1:**
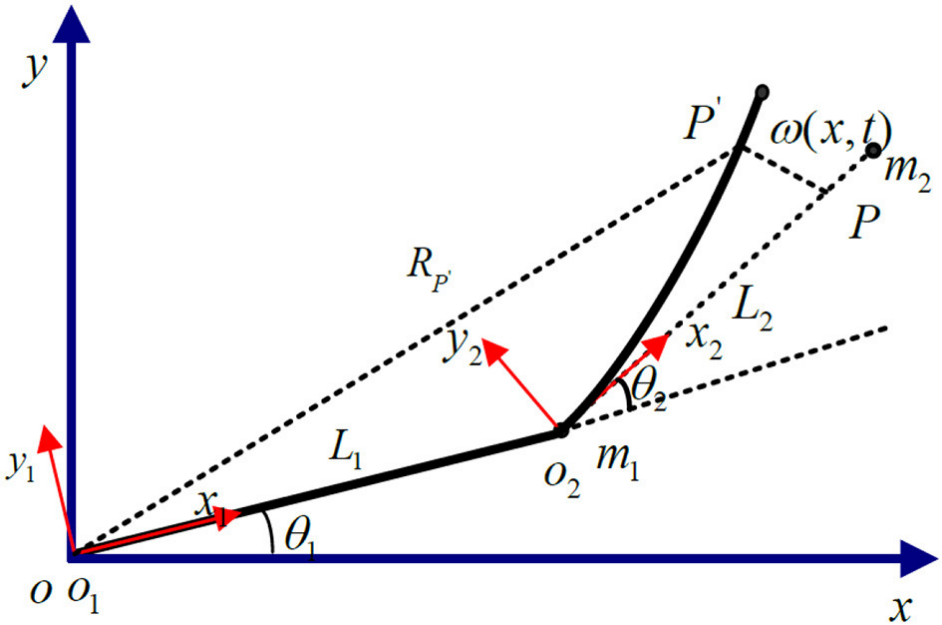
Structural diagram of the rigid-flexible robotic arm.

The research object of this paper is a two-link rigid-flexible hybrid robotic arm, composed of a rigid robotic arm, and a flexible robotic arm connected together. The rigid robotic hand is mounted on the rotating joint of the base, while the flexible robotic hand is connected to the rigid robotic hand through a motor-driven shaft. Ignoring the longitudinal deformation of the flexible robotic hand, it is assumed that the flexible robotic hand can bend freely in the horizontal direction, and the cross-section after deformation is perpendicular to the deformation axis, as shown in Figure 1.

In the Figure 1: OXY represents the inertial coordinate system fixed on the base; *o*_1_*y*_1_*z*_1_ and *o*_2_*y*_2_*z*_2_ are local coordinate systems, respectively, fixed on the rigid link robotic arm and the flexible link robotic arm base, and they rotate following the rotation of the robotic arm; θ_1_ is the angle of rotation of *o*_1_*y*_1_*z*_1_ relative to the inertial coordinate system; θ_2_ is the angle of rotation of *o*_2_*y*_2_*z*_2_ relative to o1x1y1; The lengths of the rigid arm and the flexible arm are *l*_1_ and *l*_2_, respectively; The deformation displacement of the endpoint P of the flexible robotic arm is represented by the spatial function and time function w(x, t).

As shown in Figure 1, the dimensionality reduction model of the rigid-flexible robotic arm based on the approximate inertial manifold can be represented as (Xu et al., 2021a):


(1) $$\begin{array}{l} M\left(\theta ,q\right)\left[\begin{array}{c} \ddot{\theta }\\ \ddot{q} \end{array}\right]+\left[\begin{array}{c} F_{1}\left(\theta ,q,\dot{\theta },\dot{q}\right)\\ F_{2}\left(\theta ,q,\dot{\theta },\dot{q}\right) \end{array}\right]+\left[\begin{array}{c} E_{1}\dot{\theta }\\ E_{2}\dot{q}+Kq \end{array}\right]=\left[\begin{array}{c} u\\ 0 \end{array}\right] \end{array}$$

Where:

$\theta ={\left[\theta_{1},\theta_{2}\right]}^{T}$: The generalized joint angle.

$q={\left[q_{1},0,0\right]}^{T}$: The vibration mode.

*M*(θ, *q*): The generalized inertia matrix.

*K* = diag(*k*_1_, *k*_2_, *k*_3_): Stiffness matrix.

*u*: Joint input force and torque.

${\left[F_{1}\left(\theta ,q,\dot{\theta },\dot{q}\right)\;F_{2}\left(\theta ,q,\dot{\theta },\dot{q}\right)\right]}^{T}$: Non-linear term.

*E*_1_: A positive definite damping matrix. *E*_2_: Structural damping matrix.


(2) $$\begin{array}{l} F_{1}\left(\theta ,q,\dot{\theta },\dot{q}\right)=\left[\begin{array}{c} J_{1}\left(\theta ,q,\dot{\theta },\dot{q}\right)\\ J_{2}\left(\theta ,q,\dot{\theta },\dot{q}\right) \end{array}\right],\;F_{2}\left(\theta ,q,\dot{\theta },\dot{q}\right)=\left[\begin{array}{c} J_{3}\left(\theta ,q,\dot{\theta },\dot{q}\right)\\ J_{4}\left(\theta ,q,\dot{\theta },\dot{q}\right)\\ J_{5}\left(\theta ,q,\dot{\theta },\dot{q}\right) \end{array}\right] \end{array}$$

Write the mass matrix as a block matrix $M\left(\theta ,q\right)={\left[\begin{array}{cc} M_{\theta \theta } & M_{\theta q}^{T}\\ M_{\theta q} & M_{qq} \end{array}\right]}_{5^{*}5}$, where $M^{-1}\left(\theta ,q\right)={\left[\begin{array}{cc} H_{\theta \theta } & H_{\theta q}\\ H_{q\theta } & H_{qq} \end{array}\right]}_{5^{*}5}$, both *M*_θθ_ and *H*_θθ_ are of order 2 × 2, both *M*_θ*q*_ and *H*_θ*q*_ are of order 2 × 3, *H*_*qθ*_ is of order 3 × 2, and *M*_*qq*_ and *H*_*qq*_ are of order 3 × 3.

Multiply both sides of the equation above by *M*^−1^, and the dynamics equation can be expressed in the following form:

**Table:** 

$\ddot{\theta }=-H_{\theta \theta }\left(F_{1}\left(\theta ,q,\dot{\theta },\dot{q}\right)+E_{1}\dot{\theta }\right)-H_{\theta q}\left(F_{2}\left(\theta ,q,\dot{\theta },\dot{q}\right)+E_{2}\dot{q}+Kq\right)+H_{\theta \theta }u$	(1)
$\ddot{q}=-H_{\theta q}\left(F_{1}\left(\theta ,q,\dot{\theta },\dot{q}\right)+E_{1}\dot{\theta }\right)-H_{qq}\left(F_{2}\left(\theta ,q,\dot{\theta },\dot{q}\right)+E_{2}\dot{q}+Kq\right)+H_{\theta q}u$	(2)

## 3. Controller design

**Figure 2:**
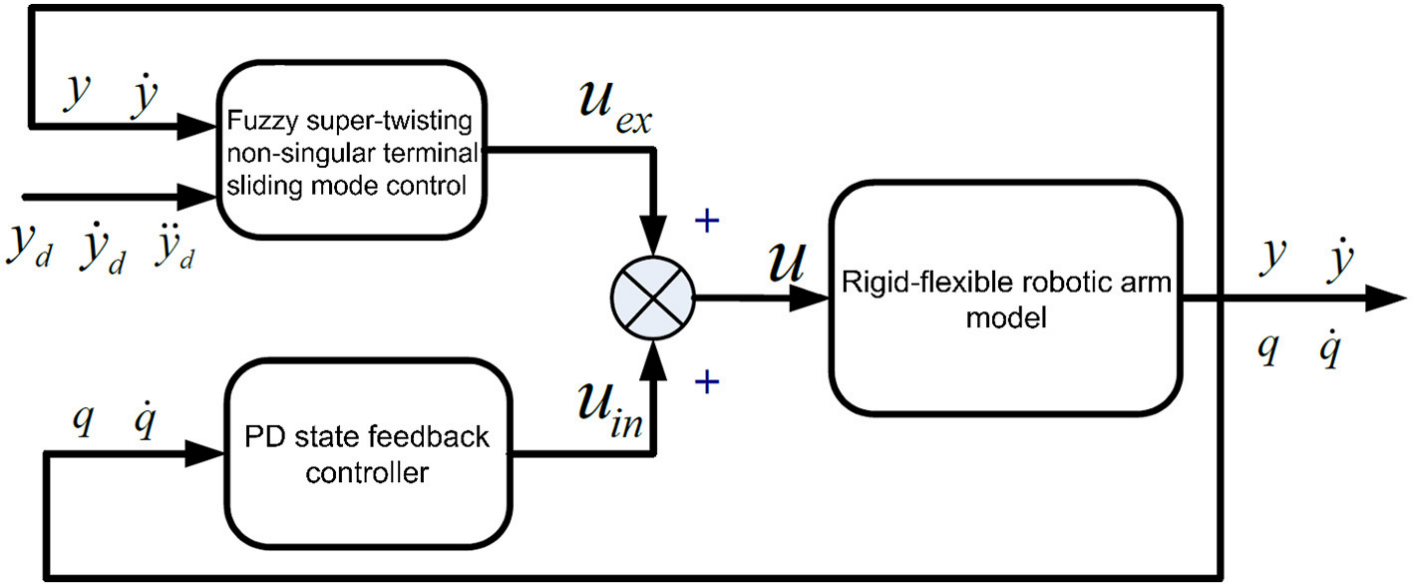
Control framework of the rigid-flexible robotic arm.

Since the rigid arm system is a minimum phase system, when establishing a closed-loop control system, the system's asymptotic stability can be ensured, making the end trajectory of the system easier to determine. In contrast, determining the end trajectory of a flexible arm is much more complex. The main challenge lies in it being a non-minimum phase system. A significant distinction between a flexible arm and a rigid arm is the presence or absence of internal dynamics. In the case of flexible arm motion, if the flexible deformation vibration is not suppressed, the arm's internal dynamics can become unstable. When adopting feedforward control, the instability of internal dynamics can easily lead to the divergence of calculated torques (Rahimi and Nazemizadeh, 2014). If feedback control is adopted, it can cause instability in the closed-loop system. Given the need to consider the dynamic model characteristics of the rigid-flexible robotic arm system, and the potential structural parameter changes, environmental changes, and component aging during operation, the controller design should firstly ensure that the input-output subsystem's design output follows the given trajectory, and secondly, ensure the stability of the internal dynamics subsystem. The proposed control structure is shown in Figure 2.

In Figure 2, *u*_*ex*_ represents the control quantity of the input-output subsystem. The control strategy adopts fuzzy super-twisting SMC. *u*_*in*_ is the control quantity of the internal dynamics subsystem, and the control strategy adopts PD (Proportional Differential) state feedback control. The overall control is composed of *u*_*ex*_ and *u*_*in*_.

### 3.1. Stabilization of the internal dynamic subsystem based on PD state feedback

According to reference (Hamdi et al., 2015), using PD state feedback to stabilize the internal dynamic subsystem ensures the convergence of the flexible deformation amount *q*.


(3) $$\begin{array}{l} u_{in}=K_{q}q+K_{\dot{q}}\dot{q} \end{array}$$

The overall input control quantity is:


(4) $$\begin{array}{l} \begin{array}{c} u=u_{ex}+u_{in}\\ =Z^{-1}\left(\alpha ,\theta ,q\right)\left({\ddot{y}}_{r}+\Lambda ė-\Gamma \left(\alpha ,\theta ,q,\dot{\theta },\dot{q}\right)+\varepsilon \cdot \mathrm{sgn}S+K_{s}S\right)\\ +K_{q}q+K_{\dot{q}}\dot{q} \end{array} \end{array}$$

Substituting Equations (7) and (9) into Equation (13) yields:


(5) $$\begin{array}{l} \{\begin{array}{l} {\dot{\text{B}}}_{1}={\text{B}}_{2}\\ \begin{array}{l} {\dot{\text{B}}}_{2}=\left(-H_{qq}K+H_{q\theta }K_{q}\right){\text{B}}_{1}+\left(-H_{qq}E_{2}+H_{q\theta }K_{q}\right){\text{B}}_{2}-\\ \;H_{q\theta }\left(F_{1}+E_{1}\dot{\theta }\right)-H_{qq}F_{2}+H_{q\theta }({\text{Z}}^{-1}(\alpha ,\theta ,q)\\ \;\left({\ddot{y}}_{r}+\Lambda \dot{e}-\Gamma (\alpha ,\theta ,q,\dot{\theta },\dot{q})+\varepsilon \cdot \mathrm{sgn}S+K_{s}S\right) \end{array} \end{array} \end{array}$$

Linearizing the internal dynamic subsystem at B_1_ = 0 and B_2_ = 0:


(6) $$\begin{array}{l} \dot{\text{B}}=\Pi_{\text{B}}\text{B} \end{array}$$

Where $\Pi_{\text{B}}=\left[\begin{array}{cc} 0 & I\\ -H_{qq0}K+H_{q\theta 0}K_{q} & H_{q\theta 0}K_{q} \end{array}\right]$ and *H*_*ij*0_ = *H*_*ij*_|_B_1_ = 0, B_2_ = 0_. By choosing appropriate control gains *K*_*q*_ and $K_{\dot{q}}$, ensuring Π_B_ is a Hurwitz matrix, it is known from Lu et al. (2018) that the internal dynamic subsystem can maintain local asymptotic stability.

### 3.2. Fuzzy super-twisting sliding mode control for input-output subsystems

#### 3.2.1. Output redefinition

Next, we redefine the output for the rigid-flexible robotic arm system, and the observed quantity of the end position output is:


(7) $$\begin{array}{l} \{\begin{array}{c} y_{1}=\theta_{1}\\ y_{2}=\theta_{2}+\alpha \cdot \frac{\omega \left(L_{2},t\right)}{L_{2}}=\theta_{2}+\frac{\alpha }{L_{2}}w\left(x,t\right) \end{array} \end{array}$$

Where the value of α is related to the output redefinition and its range is −1 < α < 1.

The end position of the rigid-flexible robotic arm is written in vector form:


(8) $$\begin{array}{l} y=\theta +D\cdot q \end{array}$$

Where:

$\text{y}={\left[{\text{y}}_{1},y_{2}\right]}^{T},\;D=\left[\begin{array}{c} 0\\ \frac{\alpha }{L_{2}}\Phi_{1}\left(L_{2}\right) \end{array}\right]$

Then, linearizing the input and output of the rigid-flexible coupled robotic arm system, we obtain the input-output subsystem:


(9) $$\begin{array}{l} \ddot{ỹ}=\tilde{\Gamma }\left(\alpha ,\theta ,q,\dot{\theta },\dot{q}\right)+\tilde{Z}\left(\alpha ,\theta ,q\right)u \end{array}$$

In the above equation:


(10) $$\begin{array}{l} \begin{array}{c} \tilde{\Gamma }\left(\alpha ,\theta ,q,\dot{\theta },\dot{q}\right)=-\left({\tilde{H}}_{\theta \theta }+\tilde{D}{\tilde{H}}_{q\theta }\right)\left({\tilde{F}}_{1}\left(\theta ,q,\dot{\theta },\dot{q}\right)+E_{1}\dot{\theta }\right)-\\ \left({\tilde{H}}_{\theta q}+\tilde{D}{\tilde{H}}_{qq}\right)\left({\tilde{F}}_{1}\left(\theta ,q,\dot{\theta },\dot{q}\right)+E_{2}\dot{q}+Kq\right) \end{array} \end{array}$$

(11) $$\begin{array}{l} \tilde{\text{Z}}\left(\alpha ,\theta ,q\right)={\tilde{H}}_{\theta \theta }+\tilde{D}{\tilde{H}}_{q\theta } \end{array}$$

Define $Ũ={\left[\begin{array}{cc} {\tilde{\text{A}}}^{T} & {\tilde{\text{B}}}^{T} \end{array}\right]}^{T}$, where $\tilde{\text{A}}={\left[\begin{array}{cc} {\tilde{\text{A}}}_{1}^{T} & {\tilde{\text{A}}}_{2}^{T} \end{array}\right]}^{T}={\left[\begin{array}{cc} {ỹ}^{T} & {\dot{ỹ}}^{T} \end{array}\right]}^{T},\tilde{\text{B}}={\left[\begin{array}{cc} {\tilde{\text{B}}}_{1}^{T} & {\tilde{\text{B}}}_{2}^{T} \end{array}\right]}^{T}={\left[\begin{array}{cc} {\tilde{q}}^{T} & {\dot{\tilde{q}}}^{T} \end{array}\right]}^{T}$. The system's state equation is as follows:


(12) $$\begin{array}{l} \{\begin{array}{c} {\dot{\tilde{\text{A}}}}_{1}={\tilde{\text{A}}}_{2}\\ {\dot{\tilde{\text{A}}}}_{2}=\tilde{\Gamma }\left(\alpha ,\theta ,\text{q},\dot{\theta },\dot{\text{q}}\right)+\tilde{\text{Z}}\left(\alpha ,\theta ,\text{q}\right)u\\ {\dot{\tilde{\text{B}}}}_{1}=\tilde{{\text{B}}_{2}}\\ {\dot{\tilde{\text{B}}}}_{2}=Ẽ\left(\theta ,\text{q},\dot{\theta },\dot{\text{q}}\right)+{\tilde{H}}_{q\theta }u \end{array} \end{array}$$

(13) $$\begin{array}{l} \tilde{E}(\theta ,q,\dot{\theta },\dot{q})=-{\tilde{H}}_{\theta q}\left({\tilde{F}}_{1}(\theta ,q,\dot{\theta },\dot{q})+E_{1}\dot{\theta }\right)-{\tilde{H}}_{qq}({\tilde{F}}_{2}(\theta ,q,\dot{\theta },\dot{q})\\ \;+E_{2}\dot{q}+Kq) \end{array}$$

In Equation (10), ${\tilde{\text{A}}}_{1}$ and ${\tilde{\text{A}}}_{2}$ form the input-output subsystem, and ${\tilde{\text{B}}}_{1}$ and ${\tilde{\text{B}}}_{2}$ form the internal dynamic subsystem:


(14) $$\begin{array}{l} \{\begin{array}{c} {\dot{\tilde{\text{A}}}}_{1}={\tilde{\text{A}}}_{2}\\ {\dot{\tilde{\text{A}}}}_{2}=ṽ \end{array} \end{array}$$

(15) $$\begin{array}{l} \{\begin{array}{c} {\dot{\tilde{\text{B}}}}_{1}={\tilde{\text{B}}}_{2}\\ {\dot{\tilde{\text{B}}}}_{2}=Ẽ\left(\theta ,\text{q},\dot{\theta },\dot{\text{q}}\right)+{\tilde{H}}_{q\theta }\cdot {\tilde{Z}}^{-1}\left(\alpha ,\theta ,\text{q}\right)\left(\hbar -\tilde{\Gamma }\left(\alpha ,\theta ,\text{q},\dot{\theta },\dot{\text{q}}\right)\right) \end{array} \end{array}$$

Where ṽℏ is the control quantity to be designed.

#### 3.2.2. Super-twisting sliding mode controller design

The sliding mode surface here is defined as in Equation (14):


(16) $$\begin{array}{l} S=e+\beta {ė}^{p/v} \end{array}$$

Where *e* = ỹ_*d*_ − ỹ represents the deviation, ỹ_*d*_ is the desired motion trajectory, β, p, ν are the sliding mode surface parameters, among which 2 > (*p*/*v*) > 1.

The super-twisting algorithm is a high-order sliding mode algorithm. It not only retains the advantages of conventional SMC but also has notable features: it can effectively suppress chattering phenomena (Gamarra-Rosado, 2000) and exhibits good tracking performance and strong robustness in the presence of bounded external disturbances (Delgado et al., 2017). Here, using the super-twisting approach, i.e., $Ṡ=-K_{1}|S{|}^{\frac{1}{2}}\mathrm{sgn}\left(S\right)-K_{2}\int_{0}^{t}\mathrm{sgn}\left(S\right)dt$, by combining Equations (8) and (14), we obtain:


(17) $$\begin{array}{l} \begin{array}{c} Ṡ=ė+\beta \frac{p}{v}{ė}^{\frac{p}{v}-1}ë=\beta \frac{p}{v}{ė}^{\frac{p}{v}-1}\left(\ddot{ỹ}-\ddot{ỹ}\right)+ė\\ =\beta \frac{p}{v}{ė}^{\frac{p}{v}-1}\left({\ddot{y}}_{d}-\tilde{\Gamma }\left(\alpha ,\theta ,q,\dot{\theta },\dot{q}\right)-\tilde{Z}\left(\alpha ,\theta ,q\right){ũ}_{ex}\right)+ė\\ =-K_{1}|S{|}^{\frac{1}{2}}\mathrm{sgn}\left(S\right)-K_{2}\int_{0}^{t}\mathrm{sgn}\left(S\right)dt \end{array} \end{array}$$

From Equations (15) and (13), the input quantity of the improved controller is:


(18) $$\begin{array}{l} \tilde{u}={\tilde{Z}}^{-1}(\alpha ,\theta ,q)(\ddot{\tilde{y}}r-{\left[\beta \frac{p}{v}{\dot{e}}^{\left(\frac{p}{v}-1\right)}\right]}^{-1}\\ \;\left(-K_{1}|S{|}^{\frac{1}{2}}\mathrm{sgn}(S)-K_{2}\int_{0}^{t}\mathrm{sgn}(S)dt-\dot{e}\right)\\ \;-\tilde{\Gamma }(\alpha ,\theta ,q,\dot{\theta },\dot{q}))+K_{q}q+K_{\dot{q}}\dot{q} \end{array}$$

#### 3.2.3. Stability proof

Theorem 1: For the aforementioned rigid-flexible robotic arm system based on the reduced model of the approximate inertial manifold, if the control parameters meet the following conditions:


$$\begin{array}{c} K_{1i}>0\\ K_{2}>\frac{K_{1i}^{2}{\left(K_{i}-K_{1i}\right)}^{2}+16K_{1i}^{2}\delta_{i}+16\delta_{i}^{2}+8K_{1i}K_{i}\delta_{i}}{8K_{i1}\left(2K_{i}-K_{1i}\right)} \end{array}$$

Where δ > 0, the designed robust super-twisting control strategy can ensure that the selected non-singular terminal sliding mode surface converges within a finite time *T*_*C*_:


$$T_{C}\leq 2\lambda_{\min}^{1/2}\left\{P\right\}\lambda_{\max}^{-1}\left\{P\right\}\lambda_{\min}\left\{Q\right\}V_{t}^{1/2}\left(0\right)$$

Proof: Firstly, insert the super-twisting control rate into the following model:


$$\ddot{ỹ}=\tilde{\Gamma }\left(\alpha ,\theta ,q,\dot{\theta },\dot{q}\right)+\tilde{Z}\left(\alpha ,\theta ,q\right)u$$

Upon arrangement, the error state equation is:


(19) $$\begin{array}{l} \begin{array}{c} ṡ=-K_{1}|s{|}^{1/2}\mathrm{sign}\left(s\right)+\psi \\ \dot{\psi }=-K_{2}\mathrm{sign}\left(s\right)+\overset{{}^{\circ}}{\varepsilon } \end{array} \end{array}$$

Where:


(20) $$\begin{array}{l} \varepsilon =\Gamma \left(\alpha ,\theta ,q,\dot{\theta },\dot{q}\right)-\tilde{\Gamma }\left(\alpha ,\theta ,q,\dot{\theta },\dot{q}\right)+K_{q}q+K_{\dot{q}}\dot{q} \end{array}$$

Let: $|\dot{\varepsilon }|\leq \delta$

For each joint, the designable Lyapunov function is:


(21) $$\begin{array}{l} V_{i}=2K_{2i}|s_{i}|+\frac{1}{2}\omega_{i}^{2}+\frac{1}{2}{\left(K_{1i}|s_{i}{|}^{1/2}\mathrm{sgn}\left(s_{i}\right)-\omega_{i}\right)}^{2} \end{array}$$

Let: $\eta_{i}={\left[|s_{i}{|}^{1/2}\mathrm{sgn}\left(s_{i}\right)\;\omega_{i}\right]}^{T}$. Rearrange equation (19) to get:


(22) $$\begin{array}{l} V_{i}=\eta_{i}^{T}P_{i}\eta_{i} \end{array}$$

Where:


$$P_{i}=\left[\begin{array}{cc} 2K_{2i}+\frac{K_{1i}^{2}}{2} & -\frac{K_{1i}}{2}\\ -\frac{K_{1i}}{2} & 1 \end{array}\right]$$

Equation (20) satisfies:


(23) $$\begin{array}{l} \lambda_{\min}\left\{P_{i}\right\}\|\eta_{i}{\|}_{2}^{2}\leq V_{i}\leq \lambda_{\max}\left\{P_{i}\right\}\|\eta_{i}{\|}_{2}^{2} \end{array}$$

(24) $$\begin{array}{l} |s_{i}{|}^{1/2}\leq \|\eta_{i}|{|}_{2}\leq \frac{V_{i}^{1/2}}{\lambda_{\min}^{1/2}\left\{P_{i}\right\}} \end{array}$$

λ_min_{*P*_*i*_} and λ_max_{*P*_*i*_}, respectively, represent the maximum and minimum eigenvalues of the matrix *P*_*i*_; $\|\eta_{i}{\|}_{2}^{2}$ represents the 2-norm of η_*i*_. The derivative of the vector η_*i*_ is:


$${\dot{\eta }}_{i}=\left[\begin{array}{c} {\dot{\eta }}_{1i}\\ {\dot{\eta }}_{2i} \end{array}\right]=\left[\begin{array}{c} \frac{1}{2|s_{i}{|}^{0.5}}{ṡ}_{i}\\ {\dot{\omega }}_{i} \end{array}\right]$$

Differentiate the Lyapunov function shown in equation (19):


$$\begin{array}{l} {\dot{V}}_{t}=2K_{2i}{\dot{s}}_{i,t}\mathrm{sgn}\left(s_{i,t}\right)+\;\omega_{i,t}{\dot{\omega }}_{i,t}+\left(K_{1i}{\left|s_{i,t}\right|}^{1/2}\mathrm{sgn}\left(s_{i,t}\right)-\omega_{i,t}\right)\\ \;\left(\frac{1}{2}K_{1i}{\left|s_{i,t}\right|}^{-1/2}{\dot{s}}_{i,t}-{\dot{\omega }}_{i,t}\right)=-2K_{1i}K_{2i}{\left|s_{i,t}\right|}^{1/2}+2K_{2i}\omega_{i,t}\mathrm{sgn}\left(s_{i,t}\right)-\\ \;K_{2i}\omega_{i,t}\mathrm{sgn}\left(s_{i,t}\right)+\omega_{i,t}{\dot{\varepsilon }}_{2i,t}\;+\left(K_{1i}{\left|s_{i,t}\right|}^{1/2}\mathrm{sgn}\left(s_{i,t}\right)-\omega_{i,t}\right)\\ \;(-\frac{1}{2}K_{1i}^{2}\mathrm{sgn}\left(s_{i,t}\right)+\frac{K_{1i}}{2}{\left|s_{i,t}\right|}^{-1/2}\omega_{i,t}\\ \;+\frac{K_{1i}}{2}{\left|s_{i,t}\right|}^{-1/2}\varepsilon_{1i,t}+K_{2i}\mathrm{sgn}\left(s_{i,t}\right)-{\dot{\varepsilon }}_{2i,t})\\ \;=\frac{1}{{\left|s_{i,t}\right|}^{1/2}}(-\frac{1}{2}K_{1i}^{3}\left|s_{i,t}\right|+K_{1i}^{2}{\left|s_{i,t}\right|}^{1/2}\mathrm{sgn}\left(s_{i,t}\right)-\\ \;\frac{K_{1i}}{2}\omega_{i,t}^{2}-K_{1i}K_{2i}\left|s_{i,t}\right|)\;+{\dot{\varepsilon }}_{2i,t}\left(-K_{1i}{\left|s_{i,t}\right|}^{1/2}\mathrm{sgn}\left(s_{i,t}\right)+2\omega_{i,t}\right) \end{array}$$

After simplification, we get:


(25) $$\begin{array}{l} {\dot{V}}_{t}=-\frac{1}{|s_{i,t}{|}^{1/2}}\eta_{t}^{T}\left[\begin{array}{cc} \frac{1}{2}K_{1i}^{3}+K_{1i}K_{2i} & -\frac{K_{1i}^{2}}{2}\\ -\frac{K_{1i}^{2}}{2} & \frac{K_{1i}}{2} \end{array}\right]\eta_{t}+{\dot{\varepsilon }}_{2i,t}\left[\begin{array}{cc} -K_{1i} & 2] \end{array}\right]\eta_{t} \end{array}$$

Let:

$A=\left[\begin{array}{cc} \frac{1}{2}K_{1i}^{3}+K_{1i}K_{2i} & -\frac{K_{1i}^{2}}{2}\\ -\frac{K_{1i}^{2}}{2} & \frac{K_{1i}}{2} \end{array}\right];\;B=\left[\begin{array}{c} 0\\ 1 \end{array}\right];C=\left[\begin{array}{c} 1\\ 0 \end{array}\right]$

Then Equation (23) can be expressed as:

${\dot{V}}_{t}=-\frac{1}{|s_{i,t}{|}^{1/2}}\eta_{t}^{T}A\eta_{t}+\frac{2{\dot{\varepsilon }}_{2i,t}}{|s_{i,t}{|}^{1/2}}|s_{i,t}{|}^{1/2}B^{T}P\eta_{t}$

Also, based on the condition: |*dε*_2,*t*_| ≤ δ_2_; the following inequality can be derived:

$\begin{array}{c} 2{\dot{\varepsilon }}_{2i,t}|s_{i,t}{|}^{1/2}B^{T}P\eta_{t}\leq {\dot{\varepsilon }}_{2i,t}^{2}|s_{i,t}|+\eta_{t}^{T}PBB^{T}P\eta_{t}\\ \leq \delta_{2i}^{2}\eta_{t}^{T}CC^{T}\eta_{t}+\eta_{t}^{T}PBB^{T}P\eta_{t} \end{array}$

Inserting into Equation (23), we obtain:

$\begin{array}{c} {\dot{V}}_{t}\leq -\frac{1}{|s_{i,t}{|}^{1/2}}\eta_{t}^{T}\left(A+\delta_{2i}^{2}CC^{T}+PBB^{T}P\right)\eta_{t}+\\ \frac{1}{|s_{i,t}{|}^{1/2}}\eta_{t}^{T}\left[\begin{array}{cc} 2K_{2i}+\frac{K_{1i}^{2}}{2} & \frac{K_{1i}}{4}\\ \frac{K_{1i}}{4} & 0 \end{array}\right]\eta_{t}\\ {\dot{V}}_{t}\leq -\frac{1}{|s_{i,t}{|}^{1/2}}\eta_{t}^{T}Q\eta_{t} \end{array}$

Where:

$Q=\frac{K_{1i}}{2}\left[\begin{array}{cc} 2K_{2i}+K_{1i}^{2}-\left(\frac{4K_{2i}}{K_{1i}}+K_{1i}\right)\delta_{1}-\frac{K_{1i}}{2}-\frac{2\delta_{2}^{2}}{K_{1i}} & -\left(K_{1i}+1\right)\\ -\left(K_{1i}-1\right) & 1-\frac{2}{K_{1i}} \end{array}\right]$

Then:


(26) $$\begin{array}{l} {\dot{V}}_{i}=-\frac{1}{|s_{i}{|}^{1/2}}\eta_{i}^{T}Q_{i}\eta_{i} \end{array}$$

When matrix *Q*_*i*_ is positive definite, Equations (5–16) satisfies Lyapunov stability. Its range of values is:


$$\begin{array}{c} K_{1i}>0\\ K_{2}>\frac{K_{1i}^{2}{\left(K_{i}-K_{1i}\right)}^{2}+16K_{1i}^{2}\delta_{i}+16\delta_{i}^{2}+8K_{1i}K_{i}\delta_{i}}{8K_{i1}\left(2K_{i}-K_{1i}\right)} \end{array}$$

### 3.3. Fuzzy controller design

The existing fuzzy non-singular terminal SMC is mainly focused on the fuzzification of the sliding mode surface exponent. This exponent has strict parameter constraints, and the method is complex. However, this paper directly performs real-time dynamic optimization on the sliding mode surface coefficient. This fuzzy strategy is simple and easy to implement. The specific design is as follows:

(1) Fuzzification of Variables

**Figure 3:**
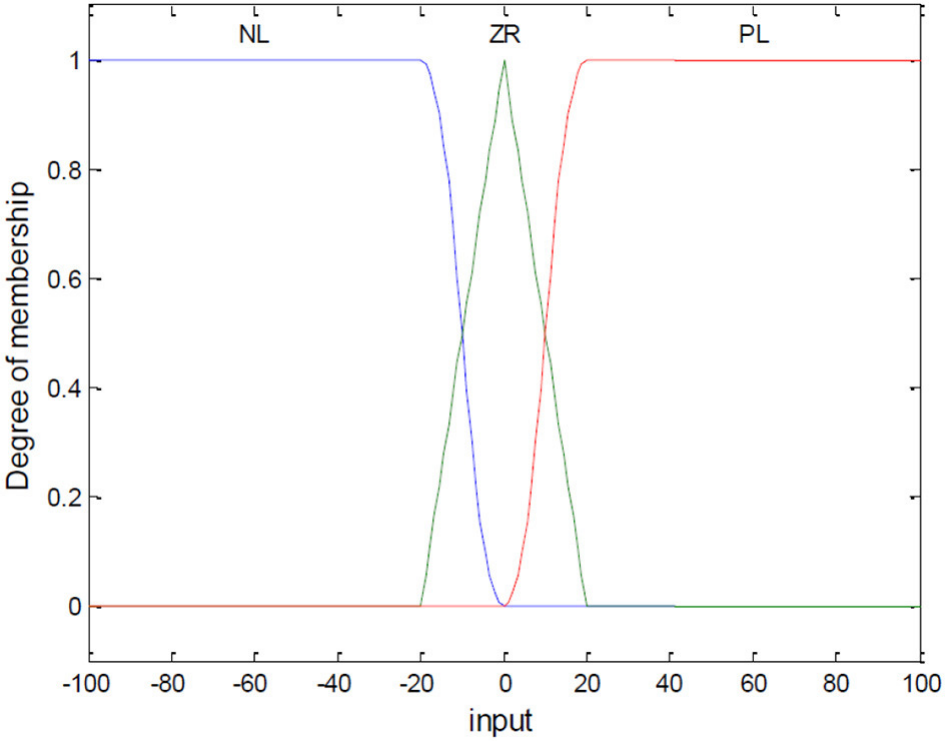
Membership function graph for input variable *S*.

**Figure 4:**
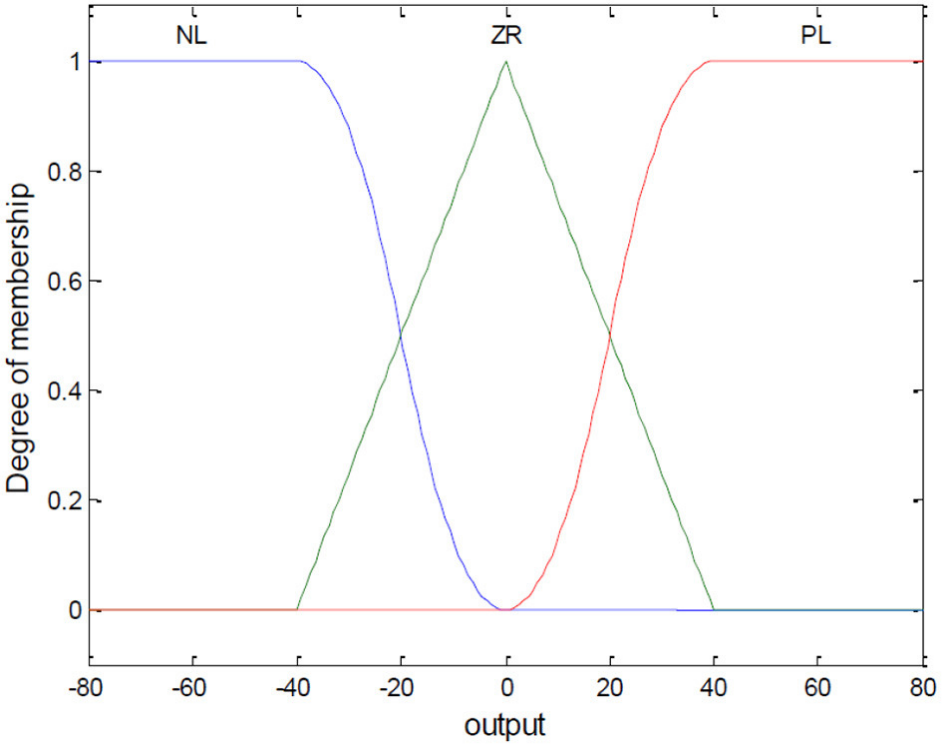
Membership function graph for output variable β.

Let S_i_ be the input of the fuzzy controller, and β_*i*_ be the output variable. Their domains are set to [−100, 100] and [−80, 80], respectively. The fuzzy variables are PL (Positive Large), ZR (Zero), NL (Negative Large). For S, the partitioned regions correspond to NL [−100, −20], ZR [−20, 20], and PL [20, 100]. For β, the regions correspond to NL [−80, −40], ZR [−40, 40], and PL [40, 80]. The membership functions are shown in Figures 3, 4.

(2) Fuzzy Control Rules

The fuzzy inference method adopts “IF-THEN.” The rules are designed as follows: If S is NL, then β is PL; If S is ZR, then β is ZR; If S is PL, then β is NL.

(3) Fuzzy Inference and Defuzzification

This step uses MATLAB's Mamdani method for inference to obtain precise values. This paper uses the centroid method, which is widely used and reflects the most comprehensive situations, as shown in Equation (25):


(27) $$\begin{array}{l} {û}^{*}=\underset{i}{\sum }{\bar{\hat{k}}}_{i}\mu_{A}\left({û}_{i}\right){û}_{i}/\underset{i}{\sum }{\bar{\hat{k}}}_{i}\mu_{A}\left({û}_{i}\right) \end{array}$$

Where i is the control quantity; û corresponds to the discretized point in the domain; and ${\bar{\hat{k}}}_{i}$ is the weighting coefficient.

## 4. Simulation analysis

### 4.1. Step response analysis

**Figure 5:**
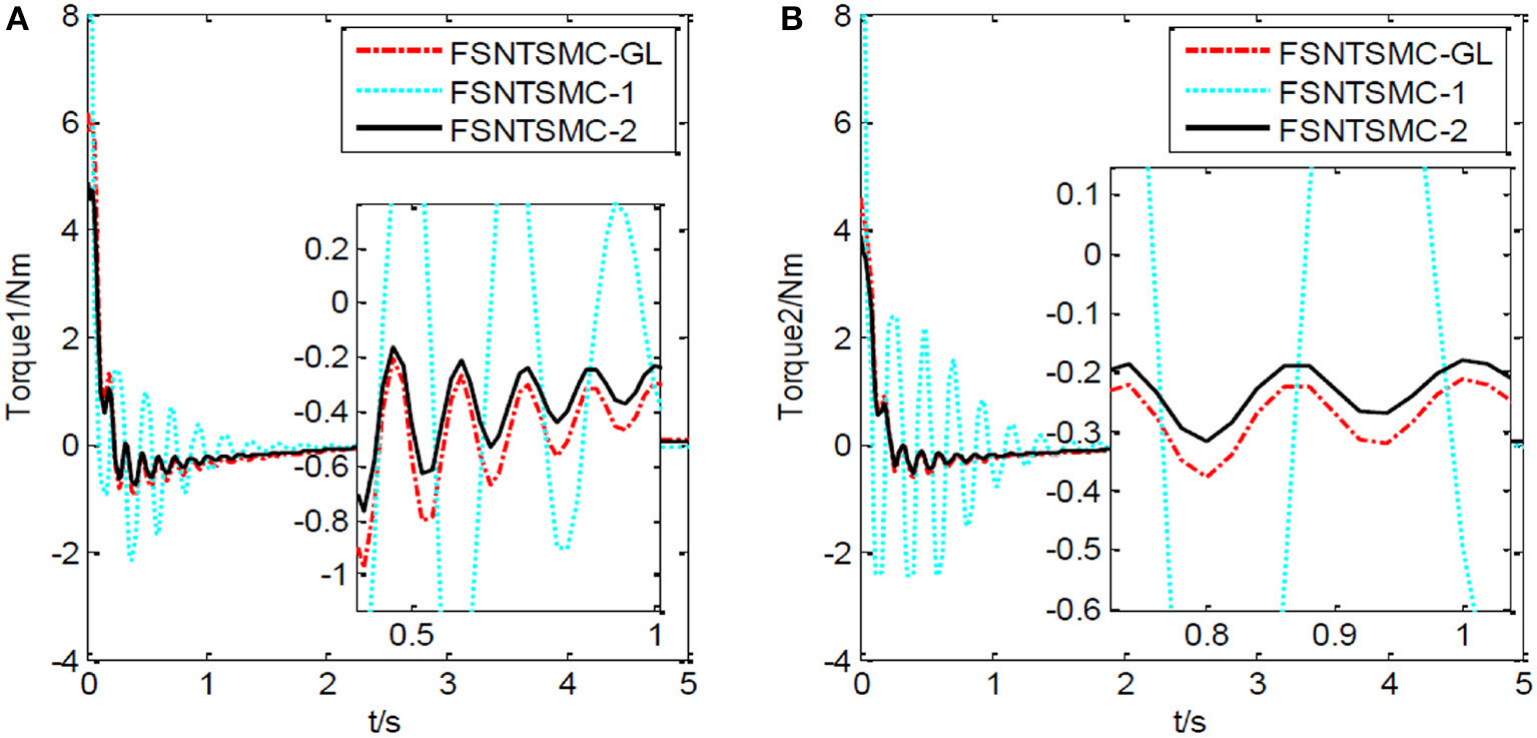
Controller's input torque: **(A)** Input torque of joint one, **(B)** input torque of joint two.

**Figure 6:**
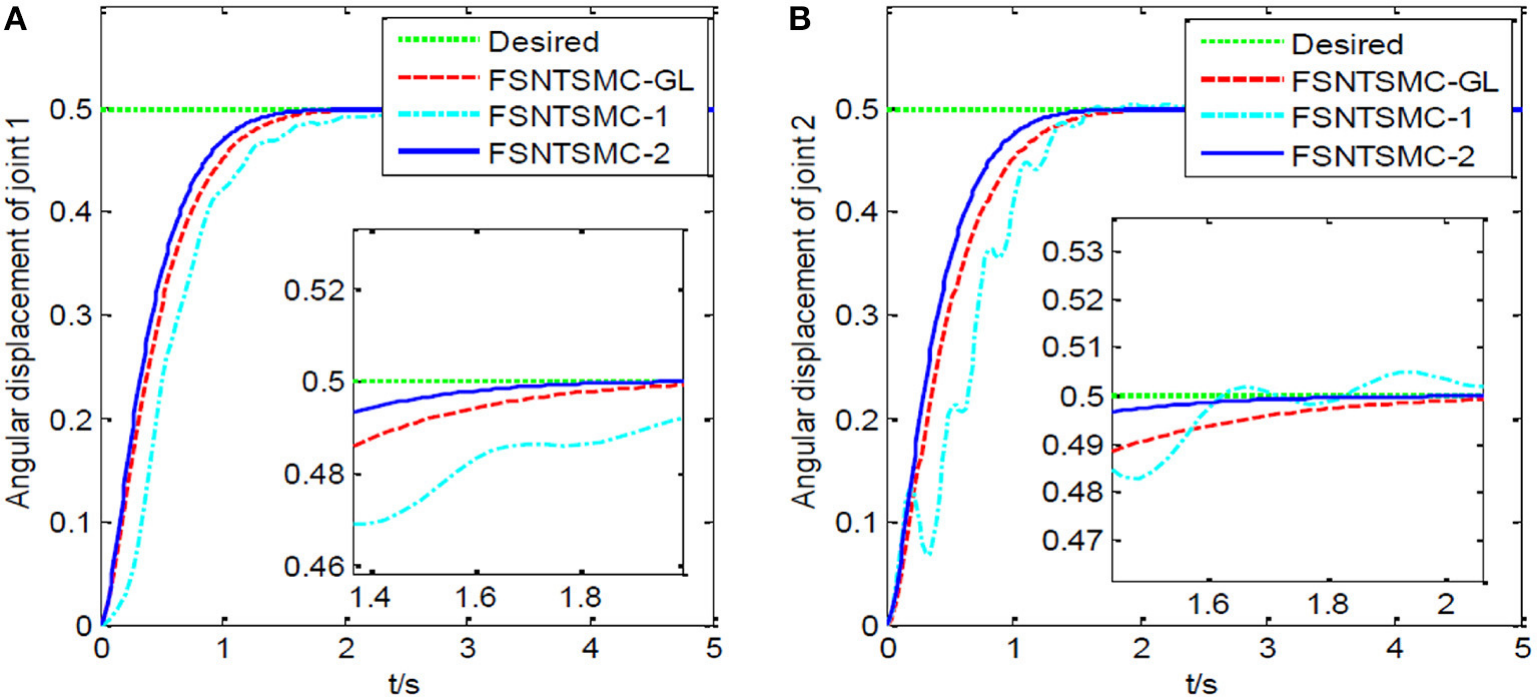
Step response of the joints: **(A)** Angular displacement of joint one, **(B)** angular displacement of joint two.

**Figure 7:**
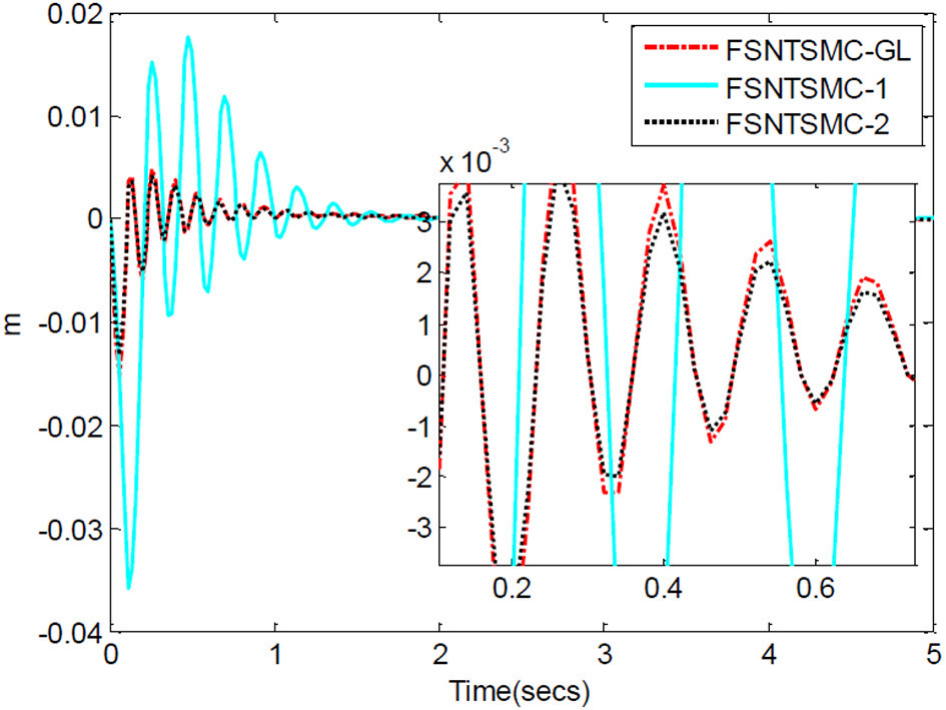
End-point vibration.

First, assume there is no external disturbance in the system and the modeling error is 0. The given step signal is ${\text{y}}_{d}={\left[\begin{array}{cc} 0.5 & 0.5 \end{array}\right]}^{T}$ (unit: rad). The parameters chosen for the second-order modal model are α = 0.65, $\;p=\left[\begin{array}{cc} 9 & 9 \end{array}\right]$, $\;v=\left[\begin{array}{cc} 7 & 7 \end{array}\right]$, *K*_1_ = diag(30, 30), and *K*_2_ = diag(9, 9), with a sampling period of 2 ms. The parameters chosen for the first-order modal model are $\alpha =0.69,\;p=\left[\begin{array}{cc} 7 & 7 \end{array}\right],\;v=\left[\begin{array}{cc} 5 & 5 \end{array}\right],\;K_{1}=\mathrm{diag}\left(22,22\right),\;K_{2}=\mathrm{diag}\left(5.6,5.6\right)$ with a sampling period of 2 ms as well. The parameters chosen for the model based on the approximate inertial manifold reduction are $\alpha =0.73,\;p=\left[\begin{array}{cc} 9 & 9 \end{array}\right]$, $v=\left[\begin{array}{cc} 7 & 7 \end{array}\right],K_{1}=\mathrm{diag}\left(25,25\right),K_{2}=\mathrm{diag}\left(7,7\right)$, with a sampling period of 2 ms too. The simulation results based on MATLAB's Simulink are shown in Figures 5–7.

FSNTSMC-1: Fuzzy super-twisting non-singular terminal SMC for the first-order modal model.

FSNTSMC-2: Fuzzy super-twisting non-singular terminal SMC for the second-order modal model.

FSNTSMC-GL: Fuzzy super-twisting non-singular terminal SMC based on the reduced model of the approximate inertial manifold.

From the simulation results in Figures 5–7, it can be seen that the FSNTSMC-GL method designed based on the reduced model of the approximate inertial manifold has significantly improved dynamic performance compared to the FSNTSMC-1 method. There is no steady-state error, no overshoot, the input torque chattering has decreased by 65%, and the end's maximum amplitude has decreased by 77.8%. The simulation results verify the effectiveness of this method.

In comparison with the second-order modal model (FSNTSMC-2), the input torque chattering, the response of the two joints, and the end vibration are very close to the second-order modal model. This provides theoretical verification for the reduced model based on the approximate inertial manifold to replace the second-order modal model for subsequent control.

### 4.2. Sine wave tracking analysis

**Figure 8:**
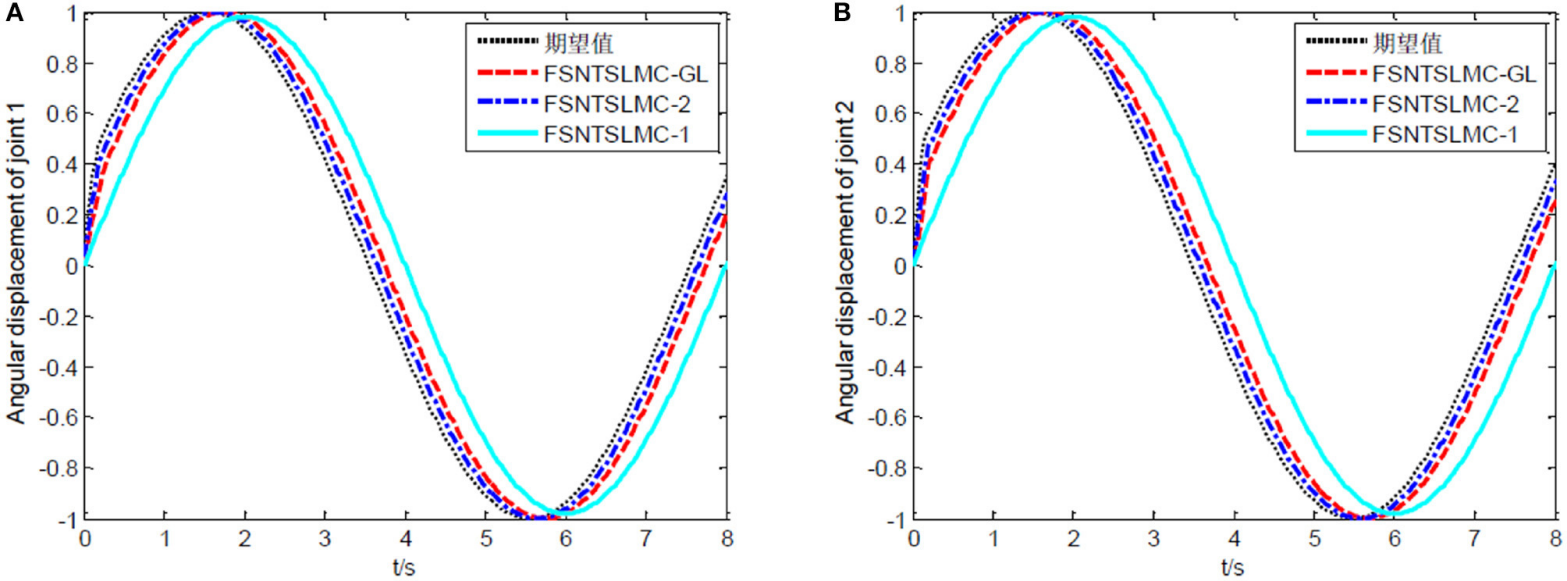
Sine tracking comparison: **(A)** Angular displacement of joint one, **(B)** angular displacement of joint two.

**Figure 9:**
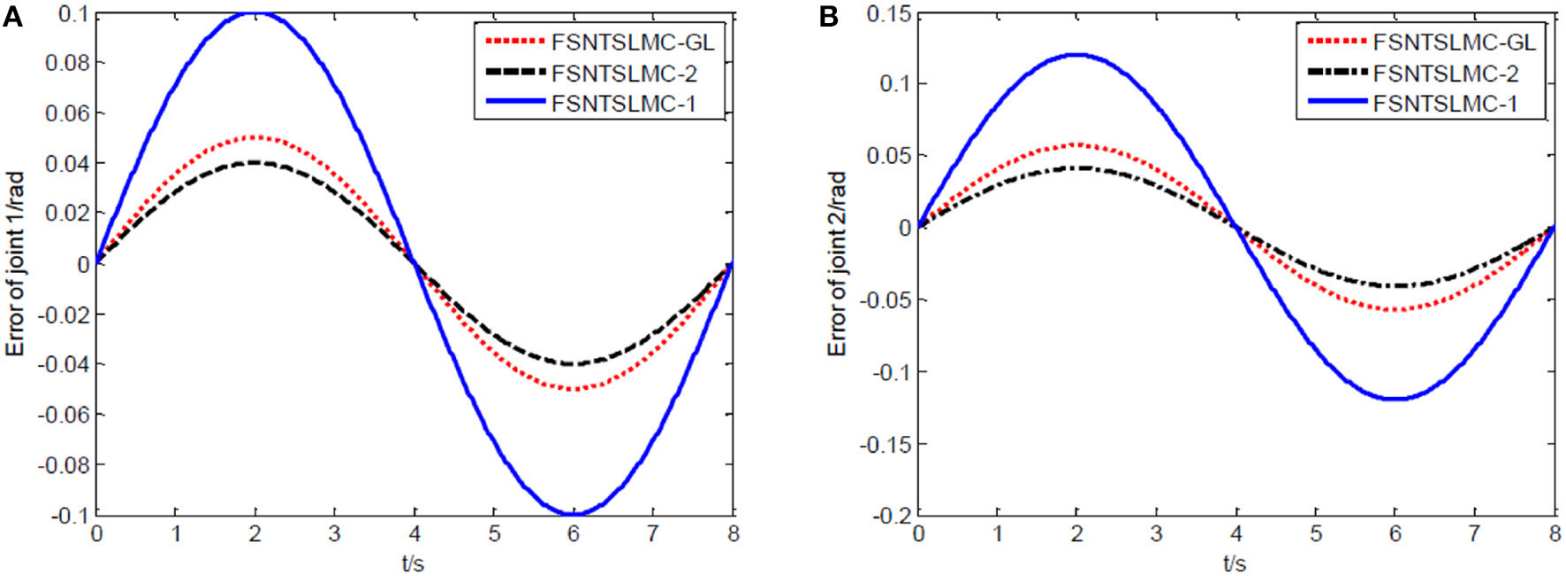
Sine tracking error comparison: **(A)** Angular displacement error of joint one, **(B)** angular displacement error of joint two.

**Figure 10:**
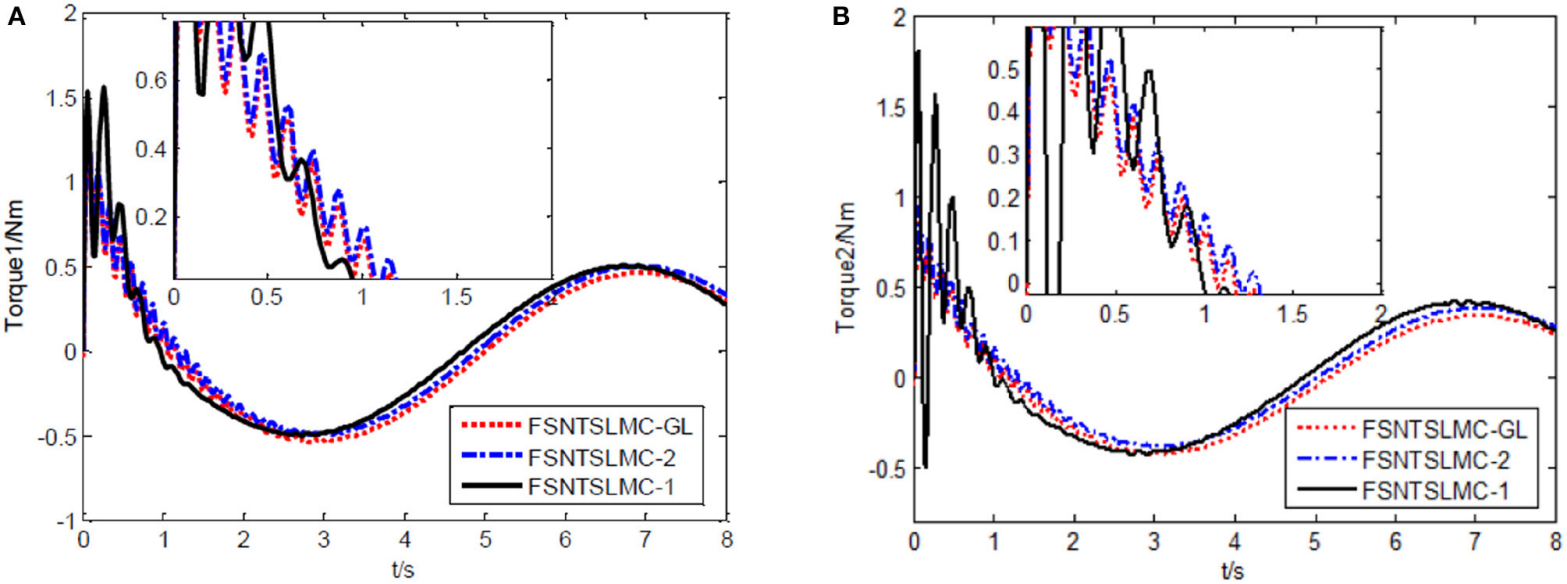
Input torque of the joints: **(A)** Input torque of joint one, **(B)** input torque of joint two.

**Figure 11:**
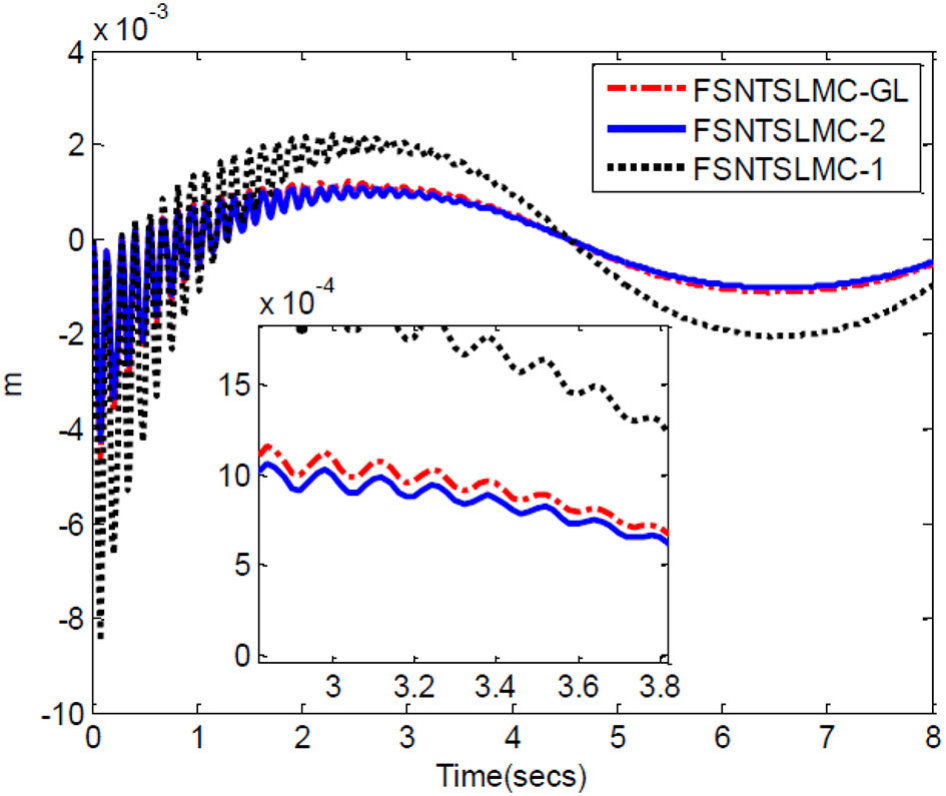
End-point oscillation.

The controller parameters chosen for sine wave tracking analysis are the same as those for step signal analysis. The sine signal input to the controller is set to $y_{d}={\left[\begin{array}{cc} \sin\left(\pi t/4\right) & \sin\left(\pi t/4\right) \end{array}\right]}^{T}$, with other parameters remaining unchanged. Similarly, assuming no external disturbance and a modeling error of 0, the simulation results based on MATLAB's Simulink are shown in Figures 8–11.

As shown in Figures 8, 9, the tracking performance, input torque chattering suppression, and end vibration suppression effects of FSNTSMC-GL and FNTSMC-2 methods are very close. Compared to the FNTSMC-1 method, the performance in all aspects has significantly improved. This further verifies that the reduced model based on the approximate inertial manifold can be used to replace the second-order modal model for controller design.

### 4.3. Robustness performance

**Figure 12:**
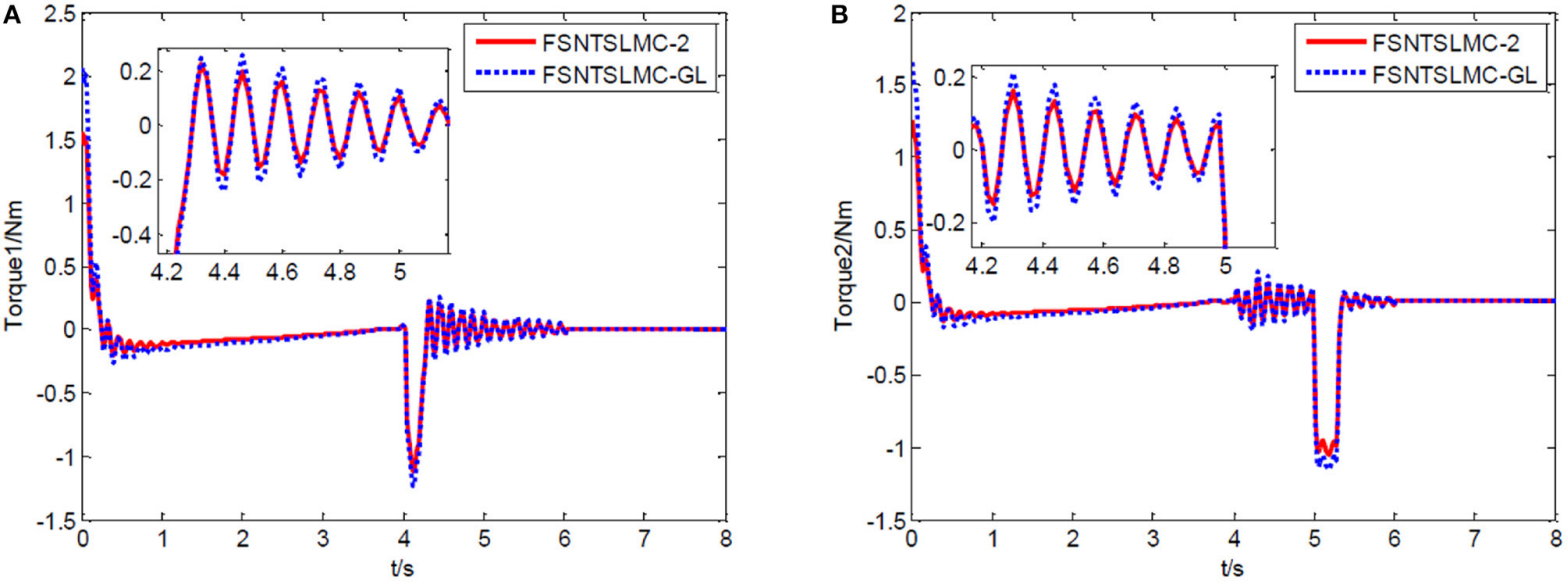
Input torque of the joints: **(A)** Input torque of joint one, **(B)** input torque of joint two.

**Figure 13:**
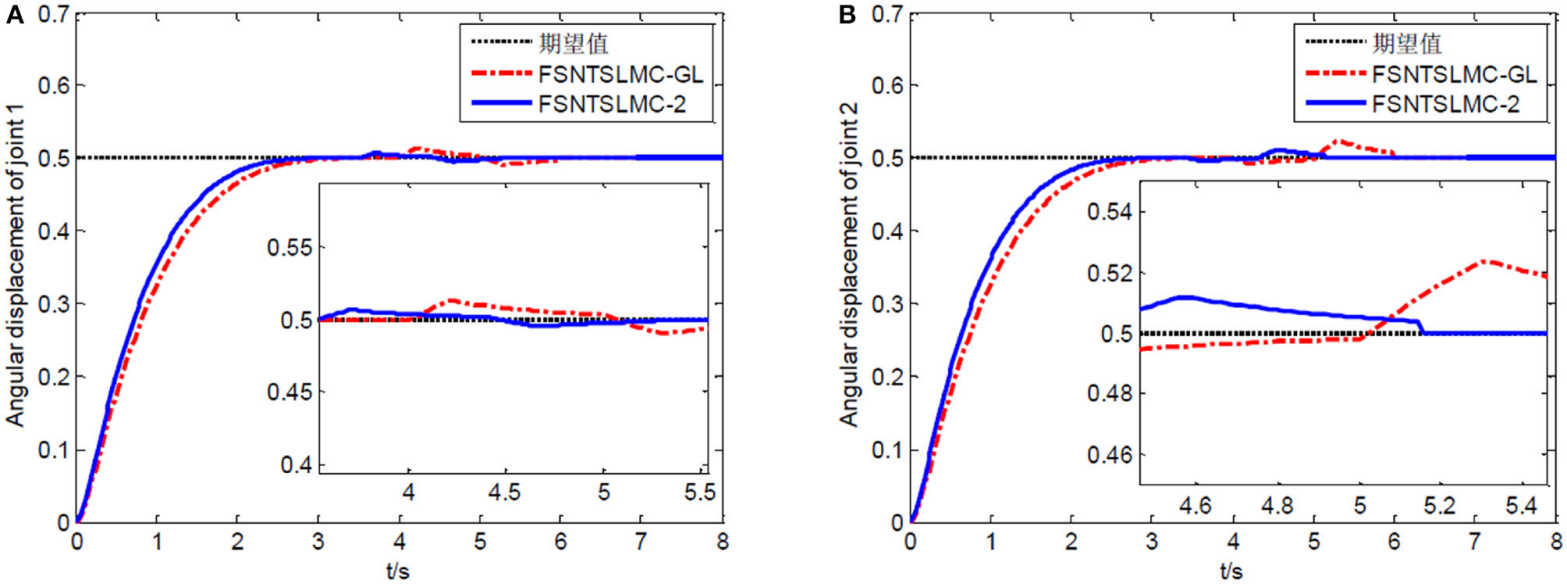
Step response of the joints: **(A)** Angular displacement of joint one, **(B)** angular displacement of joint two.

**Figure 14:**
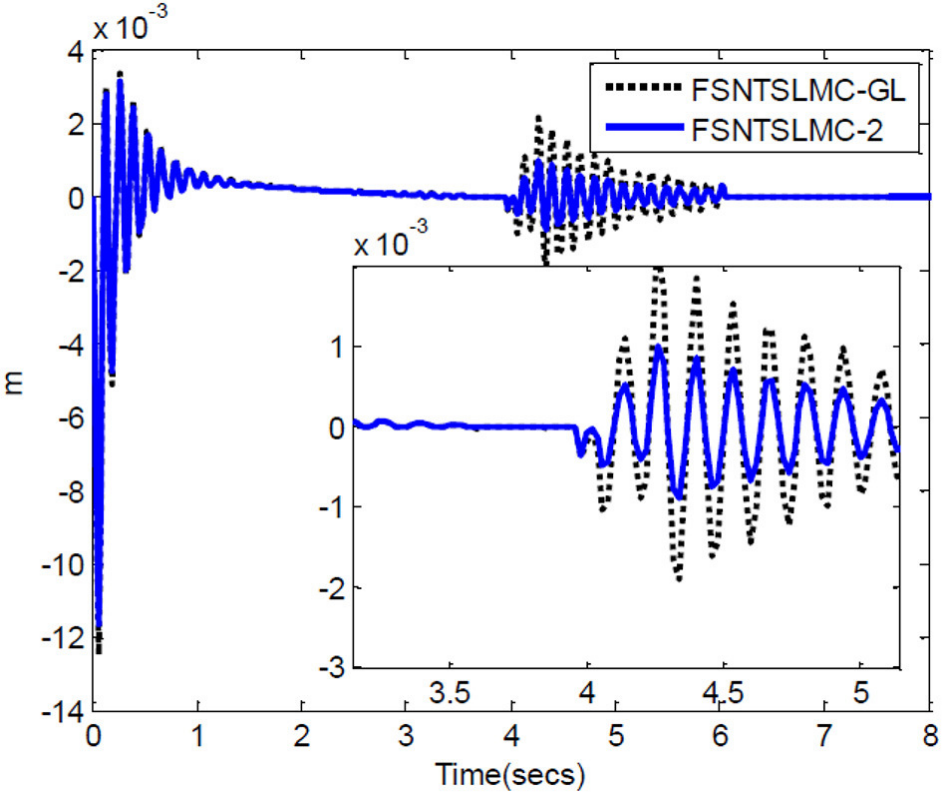
End-point vibration.

From the above step response and sine wave tracking results, it is evident that the fuzzy super-twisting sliding mode controller based on the reduced model of the approximate inertial manifold has a control quality almost equal to the second-order modal model, and significantly superior to the first-order modal model. The simulation analyses of the designed controller mentioned above were all based on the assumption of no external disturbances and zero modeling error. However, during the long-term operation of the rigid-flexible robotic arm system, hardware structure changes due to motion friction, component aging, or environmental factors may cause system structural parameter variations. This necessitates a further analysis of the robustness performance of the controller based on the reduced model of the approximate inertial manifold and a comparison with the second-order modal model. The detailed comparison is as follows:

Firstly, an analysis of the controller's robustness performance under external disturbances. The controller parameter selection remains unchanged. A 2 Nm disturbance for joint one is introduced at the 4th second and lasts for 0.2 s, while a 2 Nm disturbance for joint two is introduced at the 5th second and lasts for 0.3 s. The simulation results are shown in Figures 12–14.

From the simulation results in Figures 12–14, it can be observed that FSNTSMC-GL has strong robustness performance under disturbances, very close to the FSNTSMC-2 method. This further validates the effectiveness of the proposed method.

**Figure 15:**
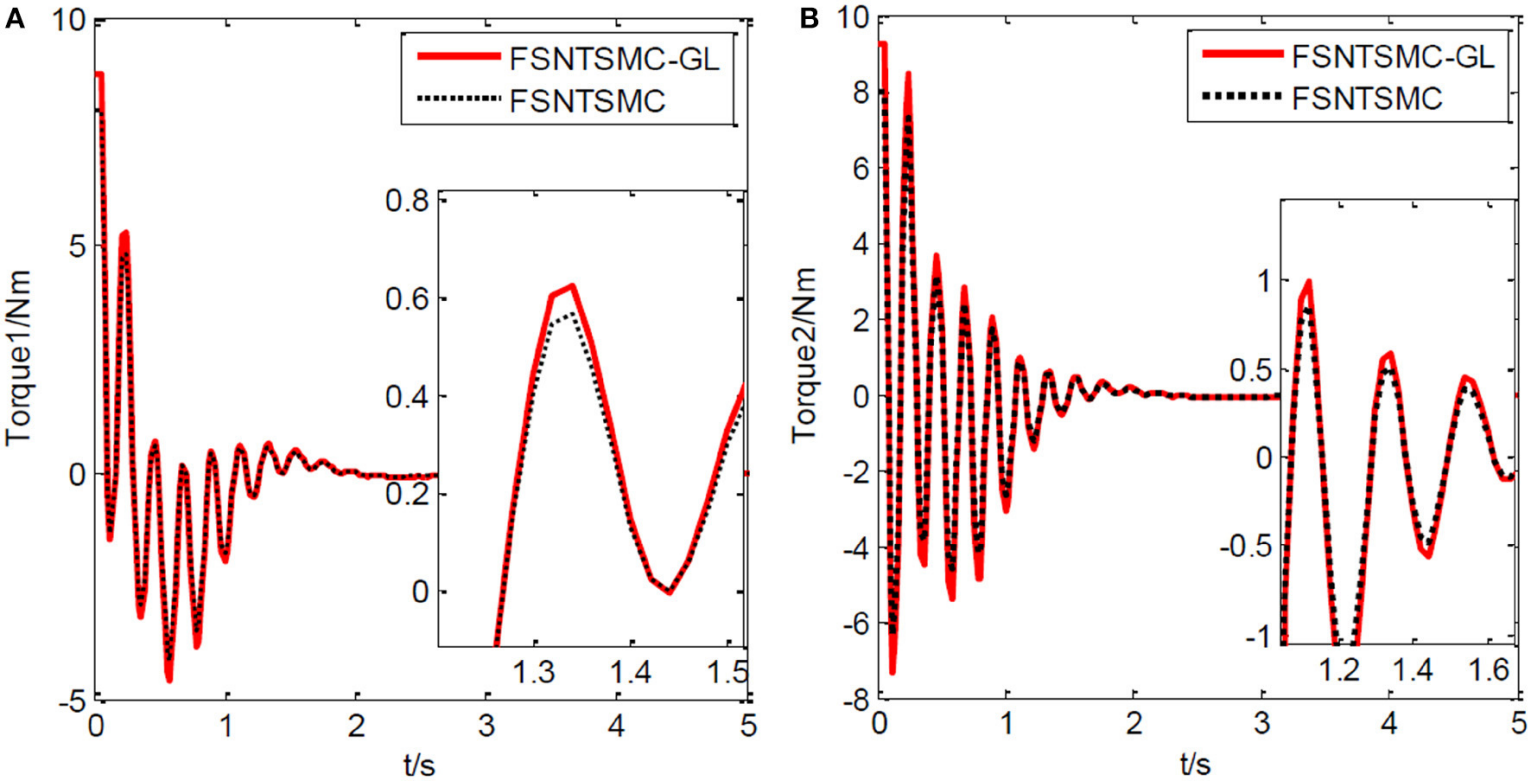
Input torque of the joints: **(A)** Input torque of joint one, **(B)** input torque of joint two.

**Figure 16:**
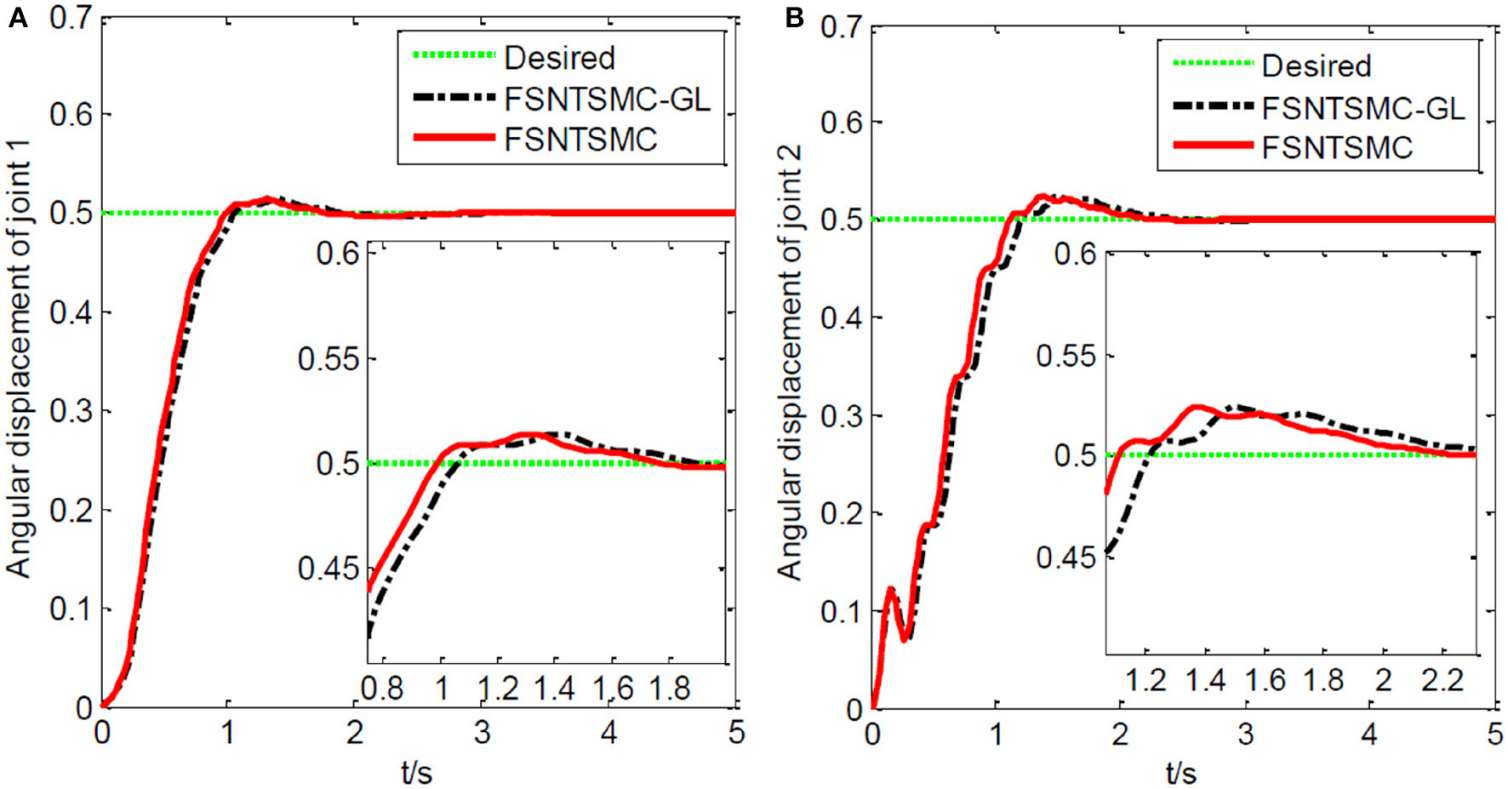
Step response of the joints: **(A)** Angular displacement of joint one, **(B)** angular displacement of joint two.

**Figure 17:**
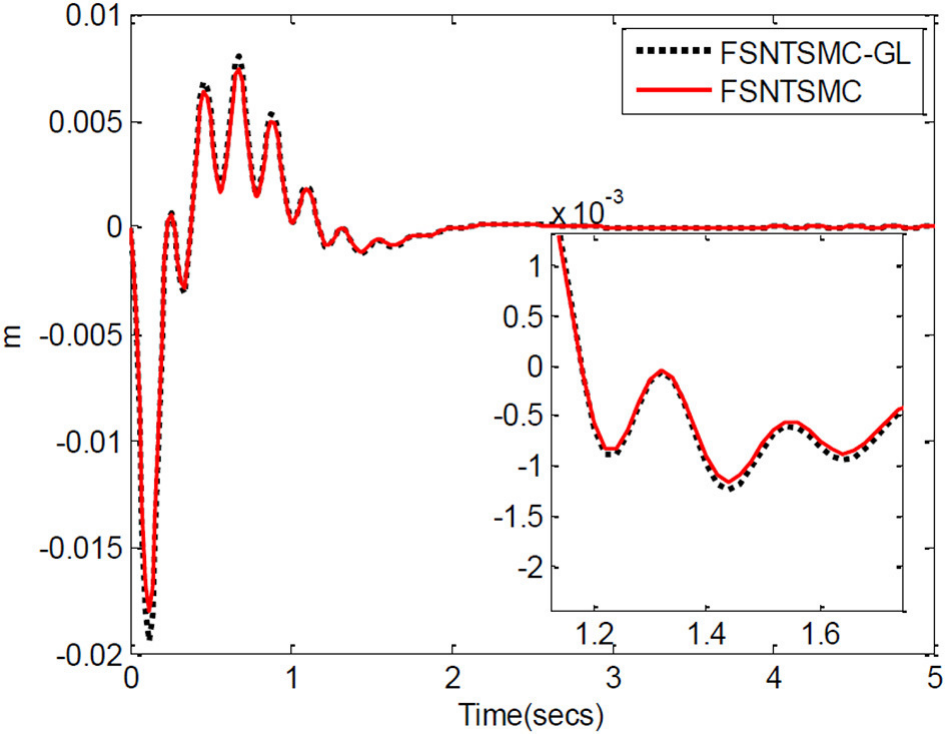
End-point vibration.

Next, a simulation analysis of the fuzzy super-twisting non-singular terminal sliding mode controller's robustness performance based on error truncation of the first-order modal model and parameter-ignored modeling errors. The controller parameter selection remains unchanged. Simulation results based on MATLAB's Simulink are shown in Figures 15–17.

From the simulation results in Figures 15–17, it is evident that FSNTSMC-GL also has strong robustness performance under error truncation and modeling errors, with performance not much different from the FSNTSMC-2 method.

## 5. Experimental verification of the fuzzy super-twisting sliding mode control for the reduced model

### 5.1. Experimental platform

**Figure 18:**
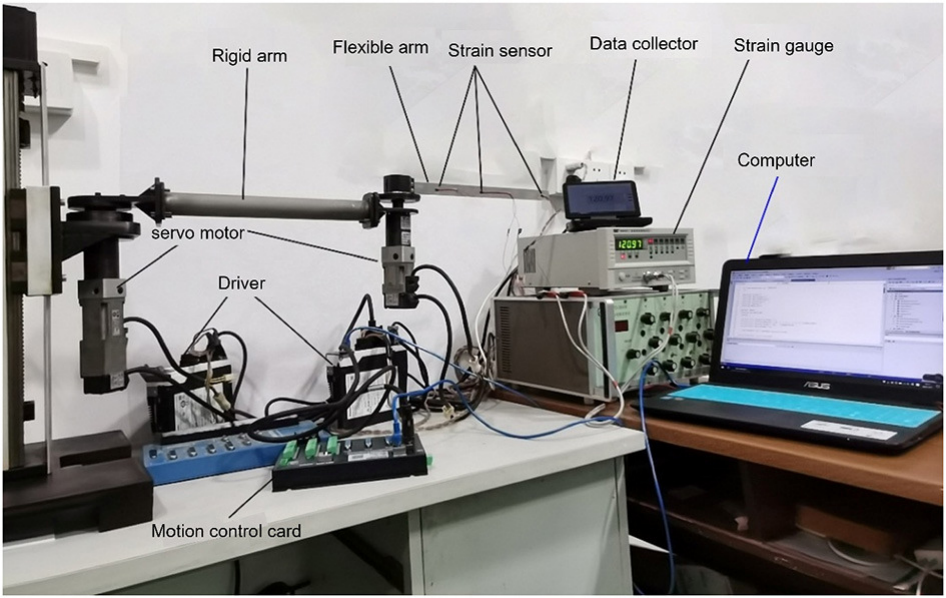
Experimental platform of the rigid-flexible robotic arm.

As showcased in Figure 18, our model verification experimental platform is physically designed to resonate with the dynamics captured by our theoretical model. Joint 1 utilizes a stepper motor and ball screw transmission, ensuring precise and smooth movements, with a grating ruler on the guide rail to measure real-time position. Joints 2 and 3, driven by servo motors with reducers, reflect the control dynamics our model emphasizes, while incremental encoders at their end capture real-time angular movements. To measure the robot's flexible arm deformation, we use strain gauges, interpreted by a dynamic strain gauge instrument, offering an exact terminal position. The basic parameters of the experimental platform are shown in Tables 1, 2.

**Table 1:** Parameters of the RK2511N+ DC resistance tester.

**Testing range**	**Accuracy**	**Open circuit voltage**	**Basic dimensions**	**Range**
10uΩ−20*K*	0.1%	<5.5*v*	330 × 220 × 198mm	2Ω/ 20Ω/200 Ω/2 *KΩ*

**Table 2:** AC servo motor parameter table.

**Motor model**	**MSMD5AZG1V**	**MSMD5AZG1U**
Rated power (W)	50	50
Rated speed (rpm)	3000	3000
Maximum speed (rpm)	5000	5000
Rated torque (Nm)	0.16	0.16
Maximum torque (Nm)	0.48	0.48
Rated line current (A)	1.1	1.1
Rotor inertia (×10^−4^kg·m^2^)	0.027	0.025

The experimental platform uses a control computer equipped with a motion control card. The industrial computer communicates in real-time with the motion control card via an Ethernet network. Resistance strain gauges are pasted at different positions on the flexible robotic arm. Displacement changes are measured through the variations in the resistance strain gauges. The control computer connects to the DC resistance tester via the RS232 standard asynchronous serial communication bus interface, recording real-time data changes in the strain gauges, ultimately obtaining the vibration displacement of the flexible arm. This verifies the accuracy of the control algorithm. The relationship between resistance change and displacement is described by the equation:


(28) $$\begin{array}{c} w\left(x,t\right)=\frac{2}{R\cdot K\cdot h}{\left[\begin{array}{c} \Phi_{1}\left(x\right)\\ \Phi_{2}\left(x\right)\\ \vdots \\ \Phi_{N}\left(x\right) \end{array}\right]}^{T}\left[\begin{array}{cccc} \Phi_{1}^{\prime \prime }\left(x_{1}\right) & \Phi_{2}^{\prime \prime }\left(x_{1}\right) & \cdots & \Phi_{N}^{\prime \prime }\left(x_{1}\right)\\ \Phi_{1}^{\prime \prime }\left(x_{2}\right) & \Phi_{2}^{\prime \prime }\left(x_{2}\right) & \cdots & \Phi_{N}^{\prime \prime }\left(x_{2}\right)\\ \vdots & \vdots & \ddots & \vdots \\ \Phi_{1}^{\prime \prime }\left(x_{i}\right) & \Phi_{2}^{\prime \prime }\left(x_{i}\right) & \cdots & \Phi_{N}^{\prime \prime }\left(x_{i}\right) \end{array}\right]\left[\begin{array}{c} \Delta R_{1}\\ \Delta R_{2}\\ \vdots \\ \Delta R_{3} \end{array}\right] \end{array}$$

R is the original resistance value of the strain gauge, Δ*R* is the resistance change caused by elongation or compression, K is the strain gauge constant (for copper-chromium alloy, the strain constant is 2), and *h* is the thickness of the flexible arm.

The control computer can obtain the generalized state quantity of flexible deformation based on the collected data and can calculate the position and instantaneous speed of the robot arm's end. Real-time control torque is obtained by transforming the control torque equation. Therefore, the servo motor system adopts a direct torque control mode. The maximum torque can be set to 0.48 Nm. Due to a reduction ratio of 40:1, the maximum torque of the final reducer output shaft is 19.2 nm. To protect the servo system and prevent the motor from being impacted by full-load torque, a safety factor of 2.0 is chosen, so the torque range of the output shaft is [−9.6 Nm, 9.6 Nm].

### 5.2. Experimental purpose and method

**Figure 19:**
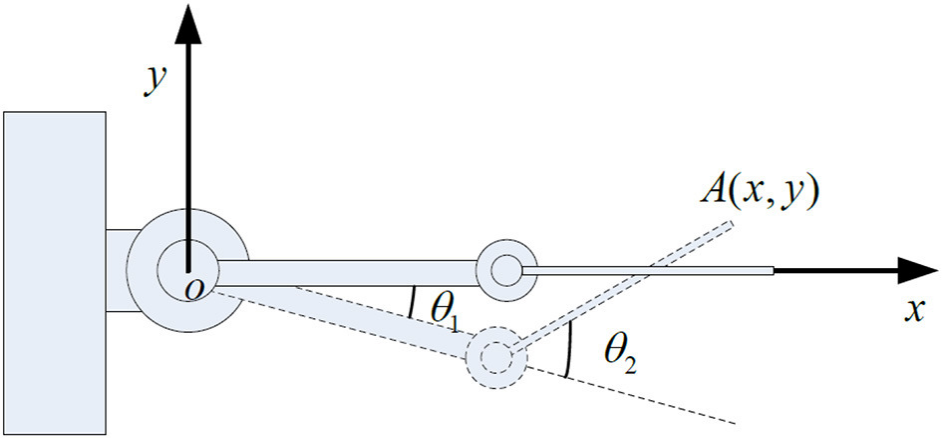
Experimental coordinate system.

**Figure 20:**
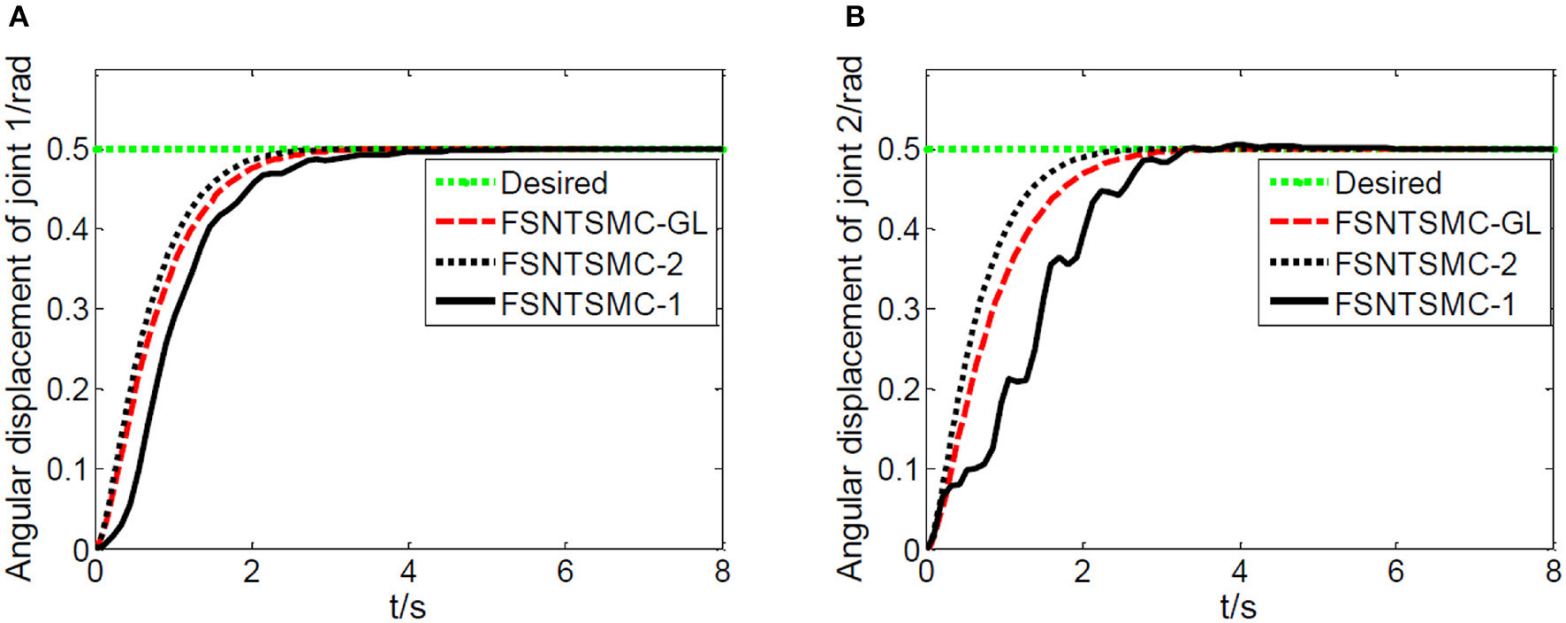
Experimental joint angular displacements: **(A)** Angular displacement of joint one, **(B)** angular displacement of joint two.

**Figure 21:**
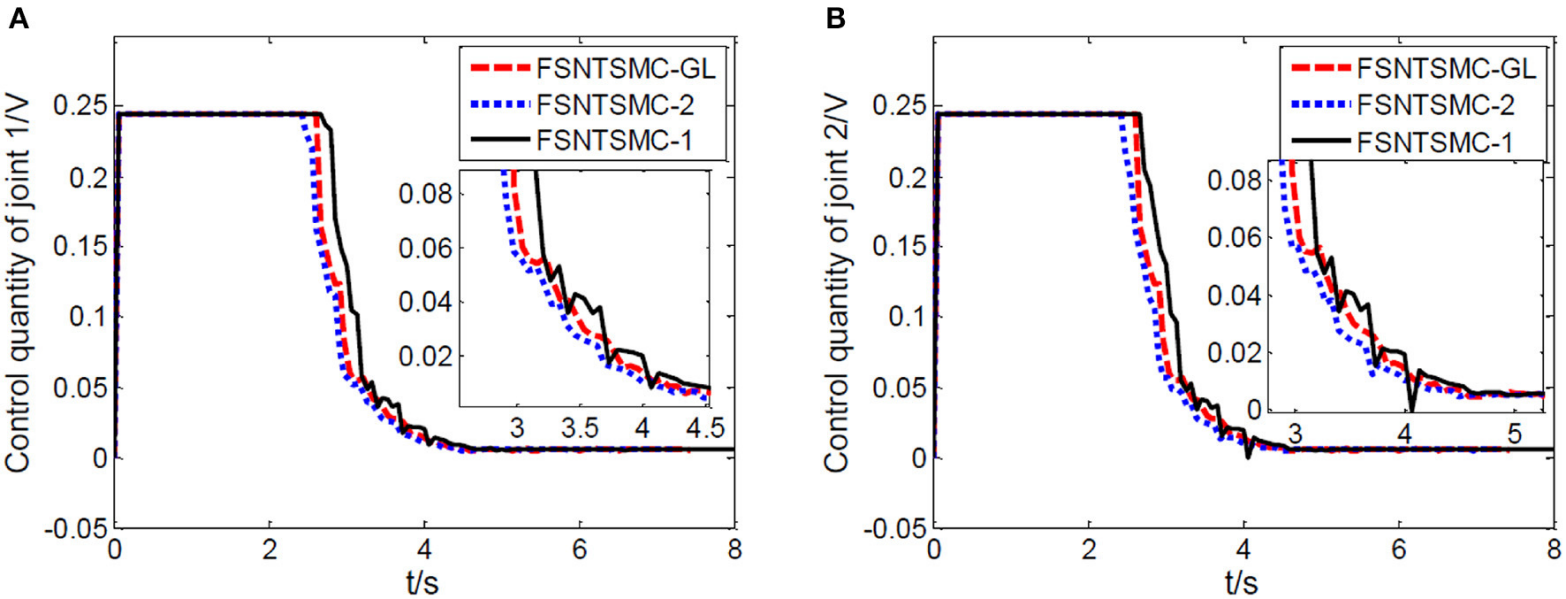
Experimental motor control value: **(A)** Control quantity of joint one, **(B)** control quantity of joint two.

**Figure 22:**
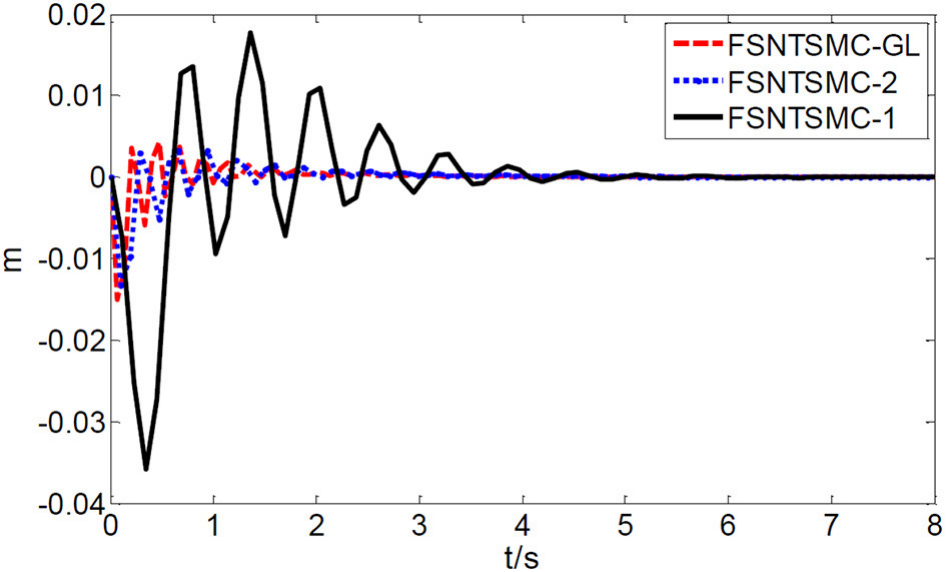
Experimental end-point deformation displacement.

As shown in Figure 19, a coordinate system is established for the experimental platform. Looking down at the experimental platform, the axis perpendicular to the guide rail plane is labeled as *x*, and the axis parallel to the guide rail plane is *y*. Therefore, the initial end position of the rigid-flexible robotic arm is (0.63, 0). In the control system we developed, a target end position coordinate is given. Through inverse calculation, the expected angles of the two joints are obtained. Using the corresponding control program, the motors are controlled with the appropriate control input until the target position is reached. During the experiment, the input control quantity of the motor is calculated by the controller program on the computer. The control card gives the motor's input, making the joints rotate. The real-time angular displacement of the two joints is obtained through the motor encoder, and the change in value of the strain gauge on the flexible arm is collected by the data collector to calculate the real-time deformation amount at the end, which is fed back to the computer for control. During the experiment, the angular displacement of the two joints, control input, and deformation amount at the end of the flexible arm are saved in real-time. We conducted an experiment using the fuzzy super-twisting sliding mode control based on the reduced-order model of the approximate inertial manifold and compared it with the direct truncation low-order modal model and the second-order modal model. The end coordinates of the experiment are (0.45 m, 0.41 m), and the experimental results are shown in Figures 20–22.

### 5.3. Analysis of experimental results

Based on the control system and experimental method described above, we conducted experimental research on the end position control of the rigid-flexible robotic arm. The experimental results, as illustrated in Figures 20–22, provide a comprehensive overview of our proposed method's performance.

From Figures 20–22, it is evident that the fuzzy super-twisting sliding mode control (FSNTSMC-GL) based on the reduced-order model of the approximate inertial manifold exhibits key attributes worth noting. Firstly, its joint response speed aligns closely with the non-singular terminal sliding mode control (FNTSMC-2) based on the second-order modal model, highlighting the comparable efficiency of our proposed method in terms of response time. Additionally, the control quantity and end vibration characteristics of FSNTSMC-GL are akin to those of FNTSMC-2, suggesting that our approach not only ensures precise control but also minimizes unintended vibrations, vital for the optimal operation of robotic arms. When contrasted with the non-singular terminal sliding mode controller (FNTSMC-1) rooted in the low-order modal model, FSNTSMC-GL demonstrates substantial improvements across all metrics, underscoring the benefits of leveraging the reduced-order model of the approximate inertial manifold, which bolsters control quality without the intricacies of advanced controller hardware implementation.

To sum up, our experimental results not only validate the theoretical claims made in the paper but also underscore the advantages of the proposed FSNTSMC-GL in real-world applications. The improved performance across response speed, control quantity, and end vibration makes it a promising alternative to existing methods, especially when hardware simplification is of paramount importance.

## 6. Conclusion

A new fuzzy super-twisting sliding mode control strategy was utilized to the reduced model of the rigid-flexible coupled robotic arm based on the approximate inertial manifold. Based on simulation and experimental results, the control quality of the low-order modal model based on the approximate inertial manifold is very close to the directly truncated second-order model. Compared to the directly truncated first-order modal model, the control quality has been significantly improved, and it has excellent tracking performance and strong robustness under bounded external disturbances. Thus, the fuzzy super-twisting sliding mode control of the rigid-flexible coupled robotic arm reduced model based on the approximate inertial manifold not only ensures higher control quality but also simplifies the controller's design.

## Data availability statement

The original contributions presented in the study are included in the article/supplementary material, further inquiries can be directed to the corresponding author.

## Author contributions

XQ: Conceptualization, Formal analysis, Funding acquisition, Methodology, Software, Writing—original draft. LX: Data curation, Investigation, Software, Validation, Visualization, Writing—review & editing. XY: Investigation, Resources, Writing—original draft.
